# METTL3-m^6^A-mediated TGF-β signaling promotes Fuchs endothelial corneal dystrophy via regulating corneal endothelial-to-mesenchymal transition

**DOI:** 10.1038/s41420-025-02384-1

**Published:** 2025-03-15

**Authors:** Jini Qiu, Xueling Zhang, Qian Shi, Yujing Yang, Rongmei Zhou, Jun Xiang, Jiayu Gu, Jianjiang Xu, Jiaxu Hong, Kun Shan

**Affiliations:** 1 Department of Ophthalmology, Eye & ENT Hospital, State Key Laboratory of Medical Neurobiology and MOE Frontiers Center for Brain Science, Fudan University, Shanghai, 200031 China; 2 NHC Key laboratory of Myopia and Related Eye Diseases, NHC, Shanghai, 200031 China; 3 Department of Ophthalmology, Yixing Eye Hospital, Wuxi School of Medicine, Jiangnan University, Yixing, 214200 Jiangsu China; 4 Shanghai Key Laboratory of Rare Disease Gene Editing and Cell Therapy, Shanghai Engineering Research Center of Synthetic Immunology, Shanghai, 200032 China; 5 Department of Ophthalmology, Children’s Hospital of Fudan University, National Pediatric Medical Center of China, Shanghai, 201102 China

**Keywords:** Cell biology, Growth factor signalling

## Abstract

Fuchs endothelial corneal dystrophy (FECD) is the leading cause of vision-threatening corneal endothelial dystrophy without pharmacologic treatments. Corneal endothelial-mesenchymal transition (cEndMT), a specific cellular phenotypic transition, is implicated in the vicious cycle in FECD pathogenesis. Here, we investigated the reversible epigenetic regulation of *N*^6^-methyladenosine (m^6^A) during cEndMT process and FECD progression. The m^6^A writer methyltransferase-like 3 (METTL3) was significantly upregulated in FECD models and induced transcriptomic hypermethylation, including *TGFB2* mRNA. METTL3 promoted the translation of hypermethylated *TGFB2* mRNA in an YTHDF1-dependent manner, resulting in upregulation of TGF-β2 protein and activation of TGF-β signaling. Intervention of METTL3 expression or catalytic activity could suppress TGF-β signaling activation, subsequently ameliorate cEndMT process and FECD progression. This study reveals unique METTL3-m^6^A-mediated mechanism in regulating cEndMT process, suggesting the prevailing role of m^6^A in cellular phenotypic transition. Targeting METTL3/m^6^A is a promising strategy for FECD treatment.

**Figure:**
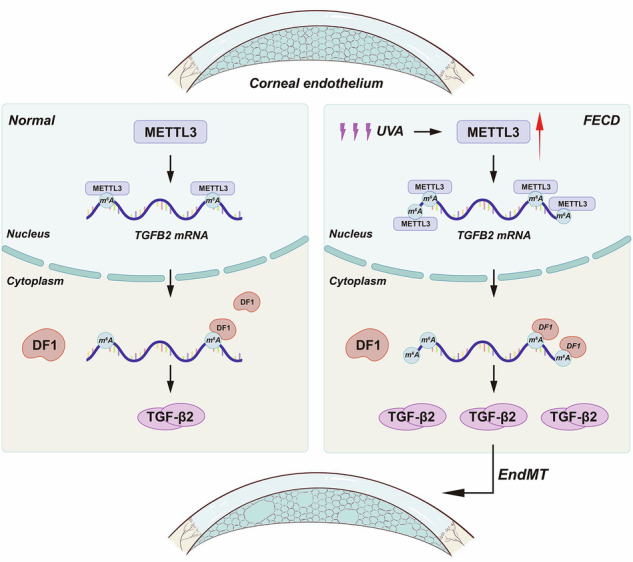
Schematic representation of METTL3-m^6^A-TGF-β signaling regulating FCED. In the context of environmental stress, METTL3 is upregulated in corneal endothelium, which in turn leads to increased m^6^A level of *TGFB2* mRNA, upregulation of TGF-β2 protein via YTHDF1 mechanism, and activation of TGF-β signaling pathway. The regulation of these mechanisms results in the progressive irreversible transition of corneal endothelial cells from their specific phenotype to a mesenchymal phenotype, which accelerates the progression of FECD.

Schematic representation of METTL3-m^6^A-TGF-β signaling regulating FCED. In the context of environmental stress, METTL3 is upregulated in corneal endothelium, which in turn leads to increased m^6^A level of *TGFB2* mRNA, upregulation of TGF-β2 protein via YTHDF1 mechanism, and activation of TGF-β signaling pathway. The regulation of these mechanisms results in the progressive irreversible transition of corneal endothelial cells from their specific phenotype to a mesenchymal phenotype, which accelerates the progression of FECD.

## Introduction

The corneal endothelium is a monolayer of orderly arranged corneal endothelial cells (CECs) and exerts indispensable functions in maintaining corneal transparency. Fuchs endothelial corneal dystrophy (FECD) is the leading cause of endogenous corneal endothelial dystrophy, characterized by alteration of cellular morphology, progressive decline of cell number, and formation of extracellular matrix excrescences (guttae), resulting in corneal blindness eventually [1]. There are no pharmacologic treatments for FECD to date, thus allogeneic corneal transplantation still remains the only way for patients to restore vision. Yet, global shortage of cornea donor supply (1 cornea available for 70 needed) and immunological rejection limit the widespread implementation of this surgery [2]. Developing effective non-surgical strategies to treat FECD is an urgent and significant unmet medical need, which relies on a better understanding of molecular mechanisms underlying FECD pathogenesis.

Environmental factors like ultraviolet (UV) light, smoking, nutrition, and hormonal effects are considered as crucial components in FECD pathogenesis [3–5]. These etiologic factors, especially UV light, could increase pathological stress in cells, affecting the onset and progression of FECD [3, 6]. In response, CECs progressively switch their specific phenotype to mesenchymal phenotype through an irreversible process entitled endothelial-mesenchymal transition (cEndMT) [7–9]. The prominent features of cEndMT process include alteration of gene expression, loss of cell-cell junctions, change in cell morphology, acquisition of cellular motility, and secretion of abnormal extracellular matrix [1]. These alternations indicate that guttae formation and abnormal cells detected in the patients are mainly attributed to the CECs that have undergone cEndMT. Recent studies have shown the guttae could further induce phenotypic switch, oxidant–antioxidant imbalance, and cellular apoptosis in surrounding CECs, emphasizing the importance of cEndMT to the vicious cycle in FECD pathogenesis [9–12]. Multiple mechanisms, containing genetic mutations (e.g., *TCF4*), aberrant expression of transcription factors (e.g., ZEB1 and SNAIL1), as well as activation of signaling pathways (e.g., TGF-β and FGF signaling pathways), might cooperate to maintain the mesenchymal phenotype of CECs, making intervention of single pathway less effective [8, 13–16].

*N*^6^-methyladenosine (m^6^A) RNA methylation, the most prominent chemical modification in messenger RNA (mRNA) and non-coding RNA in eukaryotic cells, is an emerging molecular mechanism regulating gene expression [17, 18]. m^6^A modification is formed by m^6^A methyltransferases (writers), including methyltransferase-like 3 (METTL3), methyltransferase-like 14 (METTL14), and Wilm’s tumor-associated protein (WTAP), and can be removed by m^6^A demethylases (erasers) such as fat mass and obesity-associated protein (FTO) and α-ketoglutarate-dependent dioxygenase alkB homolog 5 (ALKBH5) [19]. Identification of these m^6^A “writers” and “erasers” suggests that m^6^A RNA methylation is a dynamic and reversible process [20, 21]. Compelling evidence has demonstrated that m^6^A modification plays crucial roles in physiological processes and stress response via regulating RNA fate at the post-transcription level, for instance, mRNA stability, translation, primary microRNA processing, and RNA-protein interactions [22–25]. The regulatory function of m^6^A modification in cellular phenotypic switch has been reported in several diseases, especially cancers [26–29]. In particular, exposure of cancer cells to UV irradiation leads to increased m^6^A modification levels to facilitate UV-induced DNA damage repair and cell survival [30]. However, the role of m^6^A modification in cEndMT process or FECD pathogenesis remains poorly understood.

Here, we demonstrated METTL3 as a crucial promoter of cEndMT during FECD progression. METTL3 was significantly upregulated in FECD models, resulting in hypermethylation in the transcriptome, especially in *TGFB2* mRNA. METTL3 accelerated the translation of hypermethylated *TGFB2* mRNA in an YTHDF1-dependent manner, leading to elevated TGF-β2 protein level and activated TGF-β signaling. Intervention of METTL3 expression or catalytic activity could suppress TGF-β signaling activation, subsequently ameliorate cEndMT process and FECD progression. Our results identify a unique METTL3-m^6^A-mediated mechanism in regulating cEndMT process, providing a novel therapeutic strategy for FECD.

## Results

### Transcriptome-wide m^6^A profiling shows global hypermethylation in FECD

**Fig. 1:**
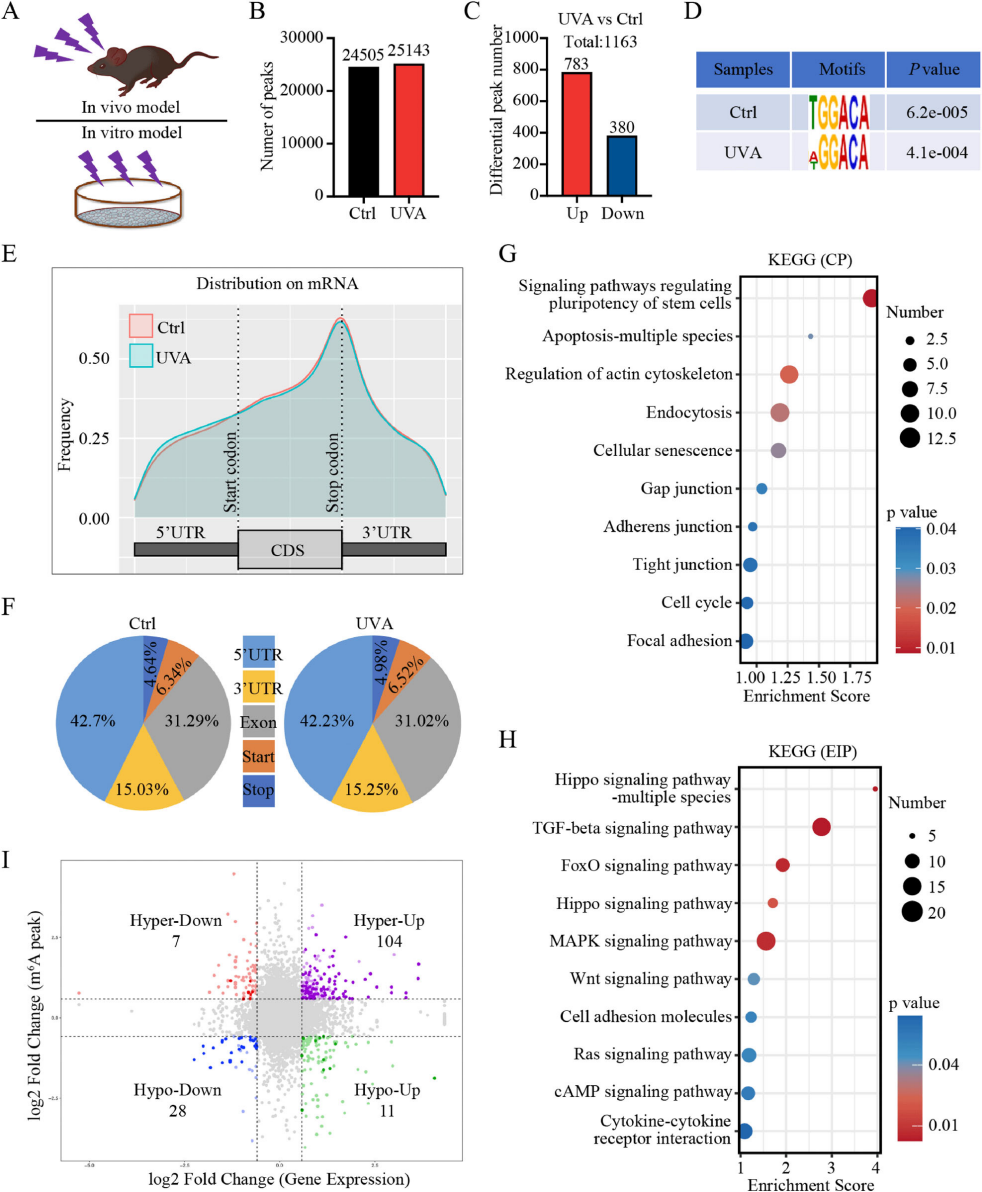
Transcriptome-wide m^6^A profiling shows global hypermethylation in FECD. **A** Schematic diagram of FECD model construction. The upper and lower panels indicate in vivo model and in vitro model, respectively. **B** Number of m^6^A peaks in control and FECD groups. **C** Differential m^6^A peaks in FECD groups compared to control groups. **D** Detection of predominant consensus motif GGAC in control and FECD groups. **E** Density distribution of m^6^A peaks across mRNA transcripts. **F** Proportion of m^6^A peak distribution across mRNA transcripts. **G**, **H** Kyoto Encyclopedia of Genes and Genomes (KEGG) pathway analysis of transcripts harbouring differential m^6^A peaks. Two subtypes are shown, including cellular processes (CP) and environmental information processing (EIP). **I** Distribution of genes with significant change in both m^6^A level (foldchange ≥ 1.5 and *P* value < 0.05) and mRNA expression level (foldchange ≥ 1.5 and *P* value < 0.05) in FECD groups compared to control groups.

Aberrant m^6^A levels suggest a regulatory role of m^6^A modification in disease process. To explore whether m^6^A levels are altered in FECD, we established UVA-induced FECD models in vivo and in vitro as previously described (Fig. 1A) [3, 31]. RNA dot blot assay was first conducted using in vivo model, and increased total m^6^A levels were detected in FECD (Supplemental Fig. 1). To identify the precise m^6^A profile in FECD, we conducted transcriptome-wide m^6^A-sequencing (m^6^A-seq) combined with RNA-sequencing (RNA-seq) assays using in vitro model. A total of 24505 (21852 in mRNA) and 25143 (22265 in mRNA) m^6^A peaks were identified in control and FECD groups, respectively, including 1163 differential m^6^A peaks, revealing the global hypermethylation in FECD (Fig. 1B, C; Supplemental Fig. 2A, B). In line with previous studies, the “GGAC” motif was highly enriched within m^6^A sites in both groups, and m^6^A peaks were significantly enriched in the vicinity of stop codon of mRNAs (Fig. 1D, E). The m^6^A distribution patterns in two groups were similar (Fig. 1F). To characterize the potential functions of these differential m^6^A peaks, Gene ontology (GO) analysis and Kyoto Encyclopedia of Genes and Genomes (KEGG) pathway analysis of transcripts harbouring differential m^6^A peaks were performed. The top 10 terms of each analysis were listed (Fig. 1G, H; Supplemental Fig. 3).

Given that m^6^A peaks were mainly enriched in mRNAs, we further classified the m^6^A-containing mRNAs into four categories. mRNA transcripts with significant increase in m^6^A peak abundance (*P* < 0.05; fold-change > 1.5) and significant upregulation in gene expression (*P* < 0.05; fold-change > 1.5) in FECD groups relative to control ones were classified into hypermethylated-upregulated category (Hyper-Up; 104). Similarly, the other three categories were hypermethylated-downregulated (Hyper-Down; 7), hypomethylated-upregulated (Hypo-Up; 11), and hypomethylated-downregulated (Hypo-Down; 28) (Fig. 1I). We also identified many significantly dysregulated mRNA transcripts without significant change in m^6^A peak abundance, and many hyper- or hypo-methylated transcripts with no significant alternation in gene expression (Fig. 1I). Collectively, these results show global hypermethylation and prevalent m^6^A alterations in mRNAs in FECD.

### m^6^A profiling reveals activation of TGF-β signaling pathway in FECD

**Fig. 2:**
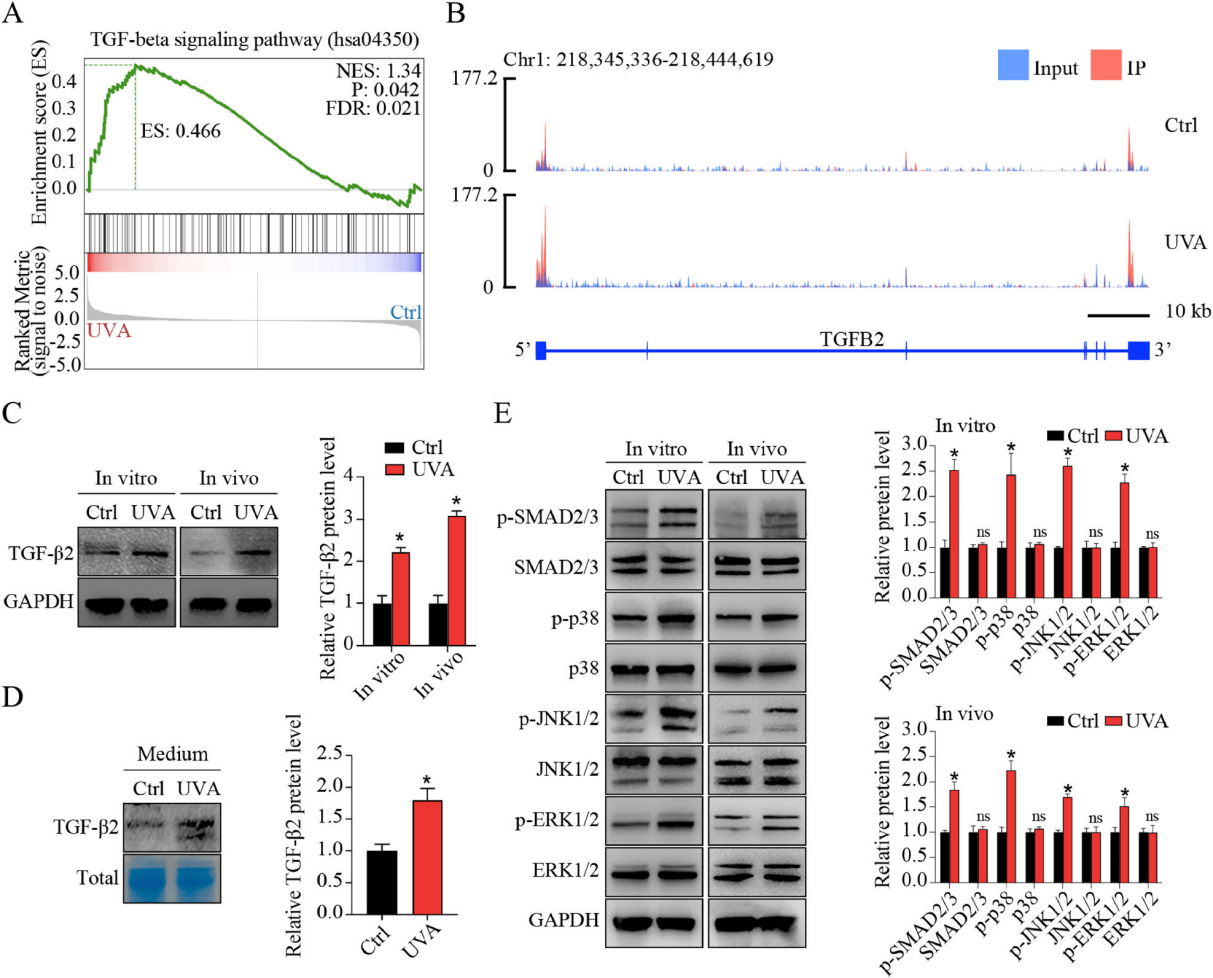
m^6^A profiling reveals activation of TGF-β signaling pathway in FECD. **A** Significant enrichment of differential m^6^A peaks in TGF-β signaling pathway shown by gene set enrichment analysis (GSEA). **B** The m^6^A abundance in TGFB2 mRNA transcripts in control and FECD groups as detected by m^6^A-seq. The m^6^A peaks have a significant increased abundance in FECD groups than in control groups, as shown by Integrative Genomics Viewer (IGV) software. **C** Western blot assay of TGF-β2 expression in vitro and in vivo. Relative quantitative expression of TGF-β2 at the protein level is shown in the right panel. **D** Western blot assay and relative quantitative analysis of TGF-β2 expression in cell culture medium. Coomassie Brilliant Blue was used to stain total proteins as the loading control. **E** Western blot assay (left panel) and relative quantitative analysis (right panel) of proteins involved in canonical SMAD signaling pathway (SMAD2/3) and non-canonical MAPK signaling pathways (p38, JNK, and ERK1/2) in vitro and in vivo. Data are presented as means ± SD from three independent experiments. *P < 0.05, ns, not significant.

On the basis of above-mentioned bioinformatics analysis, we observed that transcripts harbouring differential m^6^A peaks were mainly enriched in terms related to TGF-β signaling pathway, such as type II transforming growth factor beta receptor binding (GO:0005114), transforming growth factor beta receptor binding (GO:0005160), TGF-beta signaling pathway (ko04350), and MAPK signaling pathway (ko04010) (Fig. 1H; Supplemental Fig. 3). Gene set enrichment analysis (GSEA) also revealed significant enrichment of differential m^6^A peaks in TGF-β signaling pathway (Fig. 2A). Therefore, we analyzed the m^6^A profiles of TGF-β family. Notably, *transforming growth factor beta 2* (*TGFB2*) mRNA was included into “Hyper-Up” category. Distributions of m^6^A peaks of *TGFB2* were visualized using Integrative Genomics Viewer (IGV) software, demonstrating a significant increased m^6^A level in *TGFB2* transcript in FECD groups, especially in the 5′ untranslated region (UTR), 3′ UTR, and the vicinity of the stop codon (Fig. 2B; detail in Supplemental Fig. 4A). In addition, *TGFB1* mRNA had no significant change in m^6^A peak abundance and gene expression, while *TGFB3* mRNA showed no significant change in gene expression but decreased m^6^A peak abundance (Supplemental Fig. 4B, C).

m^6^A modification mediates the fate of mRNAs. Thus, we detected the expression of *TGFB2* and *TGFB3* at protein level. Western blot (WB) assay showed elevated TGF-β2 protein level in cells and tissues (Fig. 2C), and increased TGF-β2 secretion in cell culture supernatants in FECD groups (Fig. 2D), whereas no significant change of TGF-β3 protein was detected (Supplemental Fig. 5A, B). To explore whether TGF-β signaling was actually activated in accordance with our bioinformatics analysis, we next detected the phosphorylation of downstream mediators implicated in canonical SMAD signaling pathway (SMAD2 and SMAD3) and non-canonical MAPK signaling pathways (p38, JNK, and ERK1/2) in vitro and in vivo. Increased phosphorylated levels of these downstream mediators were detected in FECD groups, indicating the activation of TGF-β signaling (Fig. 2E). It’s worth noting that the m^6^A peak abundance of these downstream mediators was not significantly changed in FECD groups as shown by IGV software, suggesting that m^6^A modification-related TGF-β signaling activation might mainly be induced by upstream TGF-β2 (Supplemental Fig. 6A–G). Collectively, these results reveal the hypermethylation of *TGFB2* mRNA, upregulation of TGF-β2 protein, and activation of TGF-β signaling pathway in FECD.

### METTL3 activates TGF-β signaling via m^6^A modification in FECD

**Fig. 3:**
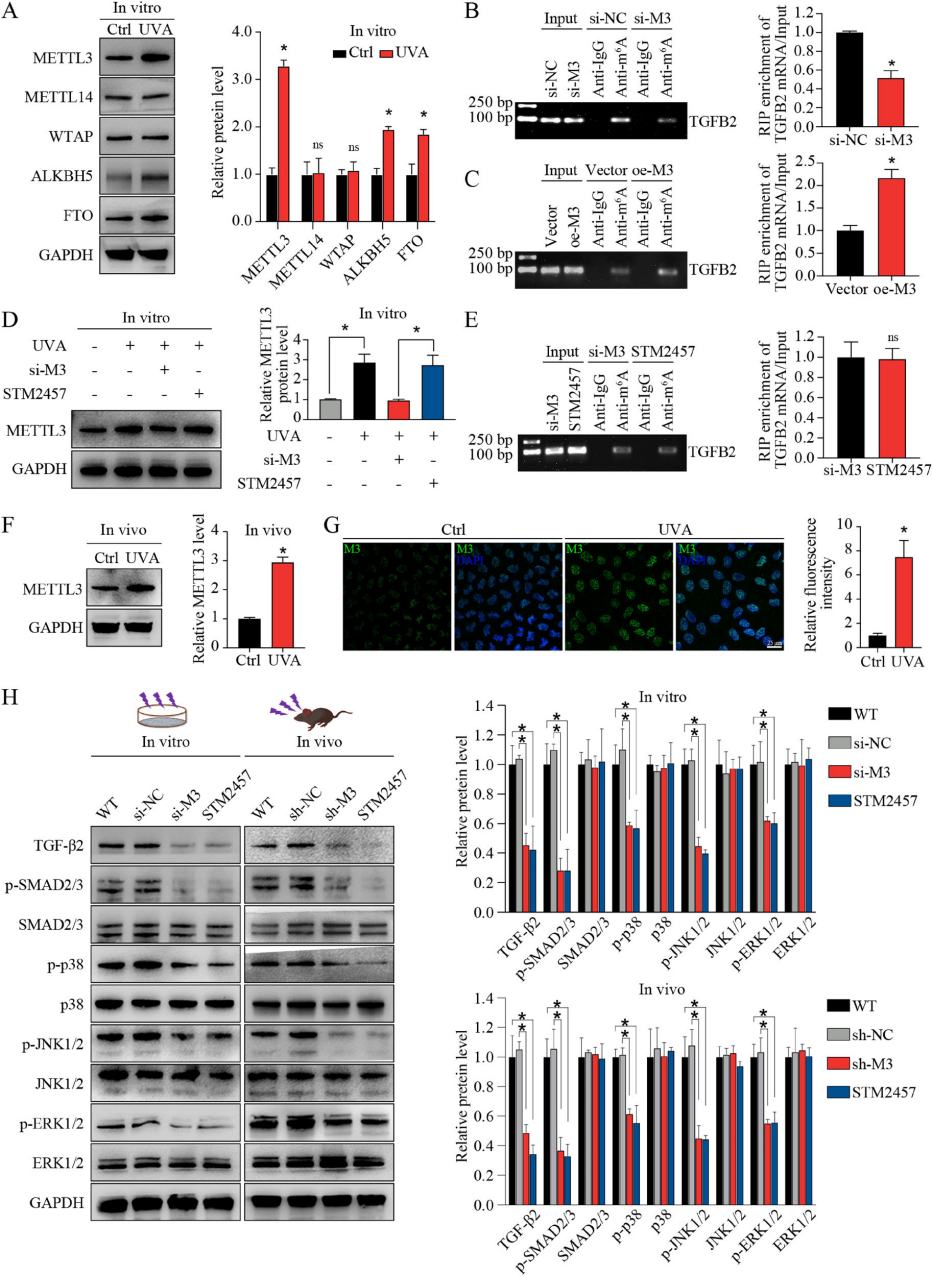
METTL3 activates TGF-β signaling via m^6^A modification in FECD. **A** Western blot assay and relative quantitative analysis of METTL3, METTL14, WTAP, ALKBH5, and FTO proteins in in vitro model. RNA immunoprecipitation combined with qRT-PCR analysis (RIP-qPCR) using anti-m^6^A antibody to evaluate the m^6^A levels of *TGFB2* mRNA in corneal endothelial cells (CECs) with METTL3-silencing (si-M3) or non-silencing (si-NC) treatment (**B**), as well as in CECs with METTL3-overexpression (oe-M3) or non-overexpression (vector) treatment (**C**). The input and anti-IgG antibody were used as positive and negative controls, respectively. Products of RT-PCR were analyzed on agarose gel and visualized with ethidium bromide staining (left panel). Enrichment of *TGFB2* mRNA was analyzed (right panel). **D** Western blot assay (left panel) and relative quantitative analysis (right panel) of METTL3 protein in CECs with different treatments in vitro. STM2457 had no influence on METTL3 expression. **E** RIP-qPCR using anti-m^6^A antibody to evaluate the m^6^A levels of *TGFB2* mRNA in CECs with si-M3 or STM2457 treatment. The input and anti-IgG antibody were used as positive and negative controls, respectively. Products of RT-PCR were analyzed on agarose gel and visualized with ethidium bromide staining (left panel). Enrichment of *TGFB2* mRNA was analyzed (right panel). The m^6^A levels of *TGFB2* mRNA were similar in CECs with si-M3 or STM2457 treatment. **F** Western blot assay (left panel) and relative quantitative analysis (right panel) of METTL3 proteins in control and FECD groups in vivo. **G** Immunofluorescence staining of METTL3 (left panel) and relative quantitative analysis (right panel) in control and FECD groups in vivo. **H** Western blot assay (left panel) and relative quantitative analysis (right panel) of TGF-β2, p-SMAD2/3, SMAD2/3, p-p38, p38, p-JNK1/2, JNK1/2, p-ERK1/2, and ERK1/2 proteins in in vitro model and in vivo model with different treatments. Data are presented as means ± SD from three independent experiments. *P < 0.05, ns, not significant.

m^6^A modification is reversibly and dynamically mediated by m^6^A modulators. To determine the modulators of increased m^6^A levels in *TGFB2* mRNA, we compared the expression patterns of several known m^6^A writers (e.g., METTL3, METTL14, and WTAP) and erasers (e.g., FTO and ALKBH5) in different groups in vitro. Quantitative reverse transcription PCR (qRT-PCR) and WB assays showed that, compared to control groups, the expression of METTL3, ALKBH5, and FTO, but not METTL14 or WTAP, was significantly upregulated in FECD groups at both mRNA (Supplemental Fig. 7) and protein levels (Fig. 3A). In consideration of the opposite roles of METTL3 and the two erasers, we speculated that METTL3 might contribute to the elevated m^6^A level in *TGFB2* mRNA. To confirm this hypothesis, we performed gene silencing (small interfering RNAs, siRNAs) targeting METTL3, ALKBH5, or FTO in in vitro FECD model, then conducted RNA immunoprecipitation combined with qRT-PCR analysis (RIP-qPCR). As expected, only METTL3 silencing could affect m^6^A level of *TGFB2* mRNA, while ALKBH5 or FTO silencing had no effect (Fig. 3B; Supplemental Fig. 8A, B). In contrast, METTL3 overexpression could increase m^6^A levels of *TGFB2* mRNA (Fig. 3C). STM2457 was reported as a selective inhibitor of m^6^A catalytic activity of METTL3, but had no effect on METTL3 expression (Fig. 3D). STM2457 application could mimic the role of METTL3 silencing in mediating m^6^A level (Fig. 3E). Increased METTL3 expression was also detected in in vivo FECD model (Fig. 3F, G). In vitro and in vivo METTL3 silencing suppressed the activation of TGF-β signaling pathway, and this phenotype could be also mimicked by STM2457 application (Fig. 3H). These results suggest that METTL3 regulates TGF-β2 expression and TGF-β signaling activation via m^6^A modification in FECD.

### METTL3-m^6^A-TGF-β signaling axis regulates cEndMT process in vitro

**Fig. 4:**
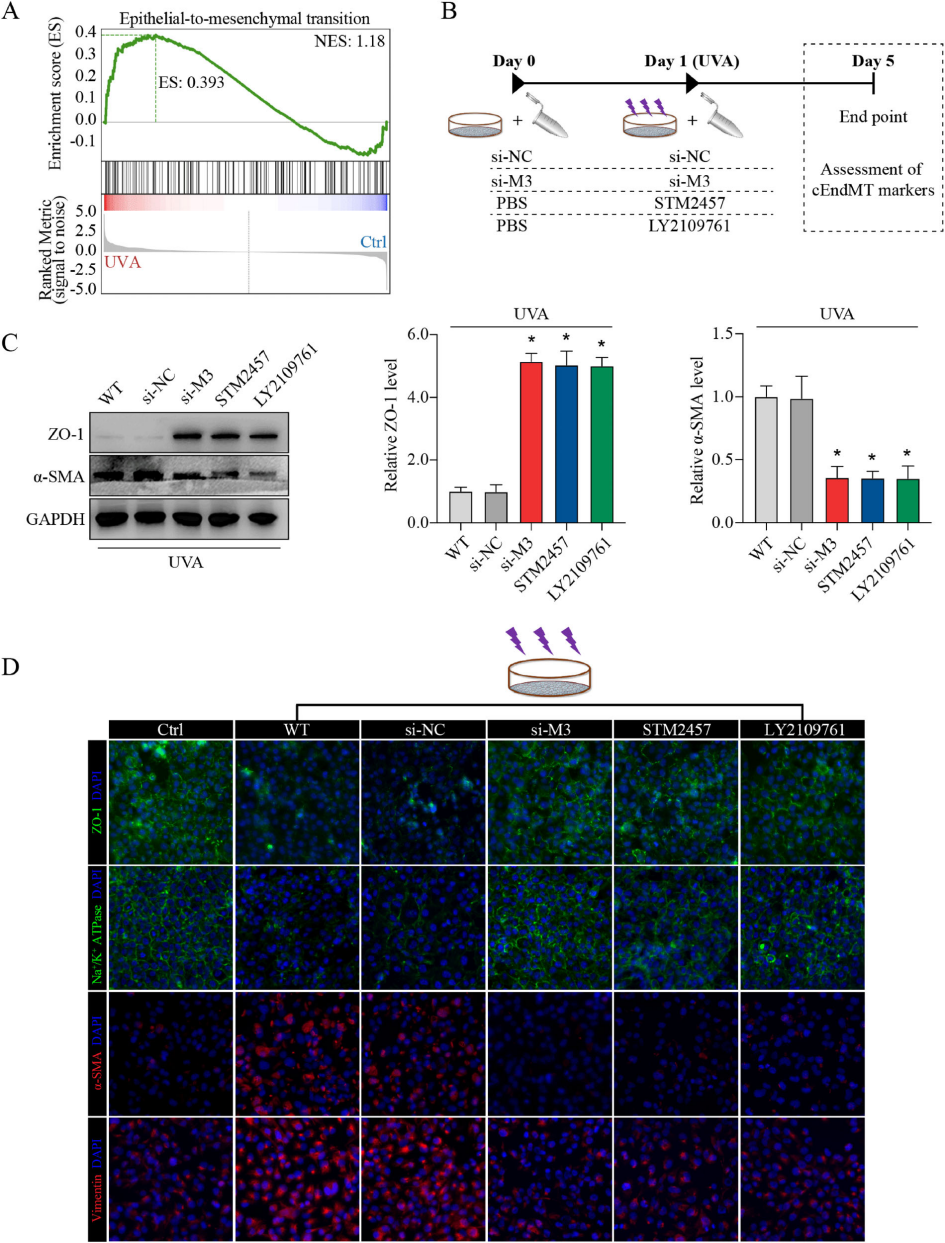
METTL3-m^6^A-TGF-β signaling axis regulates cEndMT process in vitro. **A** Enrichment of differential m^6^A peaks in terms related to phenotypic transition shown by gene set enrichment analysis (GSEA). **B** Experimental design in vitro. **C** Western blot assay (left panel) and relative quantitative analysis of ZO-1 protein (middle panel) and α-SMA protein (right panel) in in vitro model with different treatments. **D** Immunofluorescence staining of ZO-1, Na^+^/K^+^-ATPase, α-SMA, and vimentin in in vitro model with different treatments. Data are presented as means ± SD from three independent experiments. *P < 0.05 versus si-NC groups.

In FECD pathogenesis, CECs can undergo phenotypic transition to mesenchymal cells through corneal endothelial-mesenchymal transition (cEndMT) process [1]. The regulation of cell-cell junctions and actin cytoskeleton is critical to this phenotypic transition. Above-mentioned bioinformatics analysis revealed significant enrichment of differential m^6^A peaks in phenotypic transition-related terms, such as tight junction (ko04530), adherens junction (ko04520), and regulation of actin cytoskeleton (ko04810) (Fig. 1G). GSEA also showed significant enrichment of differential m^6^A peaks in terms related to phenotypic transition (Fig. 4A). Given that plenty of research has demonstrated TGF-β as the master regulator of both epithelial- or endothelial-mesenchymal transition, we speculated that METTL3-m^6^A-TGF-β signaling axis might participate in cEndMT process regulation in FECD [32].

To verify our hypothesis, we mediated METTL3 expression or catalytic activity, and detected the influence on cEndMT process in vitro (schematic diagram shown in Fig. 4B). WB assay and immunofluorescence (IF) staining confirmed the occurrence of cEndMT in FECD model, as showed by decreased expression of corneal endothelial markers (e.g., ZO-1, and Na^+^/K^+^-ATPase) and increased expression of mesenchymal markers (e.g., α-SMA and vimentin) (Fig. 4C, D; Supplemental Fig. 9A–D). Silencing of METTL3 ameliorated cEndMT process, which could be mimicked by application of STM2457, indicating the role of METTL3-mediated m^6^A modification in cEndMT process (Fig. 4C, D; Supplemental Fig. 9A–D). To confirm that the function of METTL3-mediated m^6^A modification is mainly through regulation of TGF-β signaling, we further applied LY2109761, a selective TGF-beta receptor type I/II inhibitor, to suppress the activation of TGF-β signaling [33, 34]. As expected, LY2109761 application could also mimic the role of METTL3 silencing or STM2457 application (Fig. 4C, D; Supplemental Fig. 9A–D). These results reveal that METTL3 could regulate cEndMT process via m^6^A-induced TGF-β signaling activation in vitro.

### METTL3-m^6^A-TGF-β signaling axis regulates cEndMT process and FECD progression in vivo

**Fig. 5:**
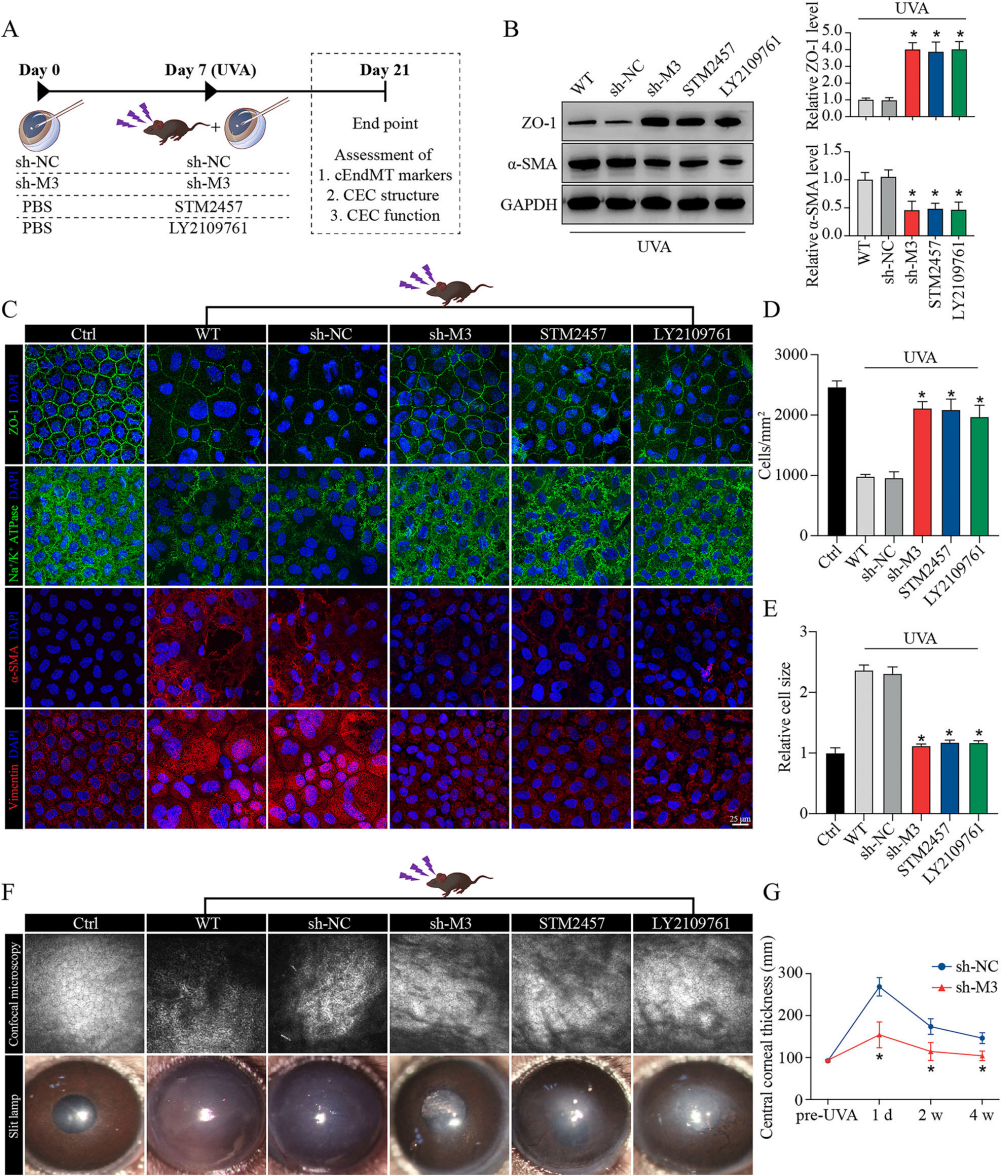
METTL3-m^6^A-TGF-β signaling axis regulates cEndMT process and FECD progression in vivo. **A** Experimental design in vivo. **B** Western blot assay (left panel) and relative quantitative analysis of ZO-1 proteins (middle panel) and α-SMA proteins (right panel) in in vivo model with different treatments. **C** Immunofluorescence staining of ZO-1, Na^+^/K^+^-ATPase, α-SMA, and vimentin in in vivo model with different treatments. Analysis of cell density (**D**) and cell size (**E**) based on ZO-1 immunostaining. **F** Observation of mouse corneas with different treatments using confocal microscopy (upper panel) and slit lamp biomicroscope (lower panel). **G** Detection of central corneal thickness using optical coherence tomography. Data are presented as means ± SD from three independent experiments. *P < 0.05 versus sh-NC groups.

To examine the biological function of METTL3-m^6^A-TGF-β signaling axis on cEndMT process in vivo, we delivered adeno-associated virus (AAV, targeting METTL3), STM2457, or LY2109761 into the anterior chamber of FECD mice. The schematic diagram of anterior chamber injection was shown (Fig. 5A). The silencing efficacy of AAV was tested (Supplemental Fig. 10). WB assay and IF staining also confirmed the role of METTL3-m^6^A-TGF-β signaling axis in regulating cEndMT process in vivo, and STM2457 application or LY2109761 usage could mimic the role of METTL3 silencing (Fig. 5B, C; Supplemental Fig. 11A–D). To investigate whether this axis could further influence FECD progression, we tested a number of indicators commonly used in clinical practice. UVA-induced FECD exhibited decreased cell density and increased cell size, which were calculated based on ZO-1 staining (Fig. 5D, E; schematic diagram shown in Supplemental Fig. 12). Confocal microscopy (CM) revealed decreased discernible cellular borders, while slit lamp biomicroscope and optical coherence tomography (OCT) revealed aggravated cornea swelling in FECD model (Fig. 5F, G). These phenotypes could be ameliorated by METTL3 knockdown, STM2457 application, or LY2109761 usage (Fig. 5D–F). These results suggest that METTL3 could regulate cEndMT process and FECD progression via m^6^A-induced TGF-β signaling activation in vivo.

### METTL3 regulates the translation of TGF-β2 in an YTHDF1-dependent manner

**Fig. 6:**
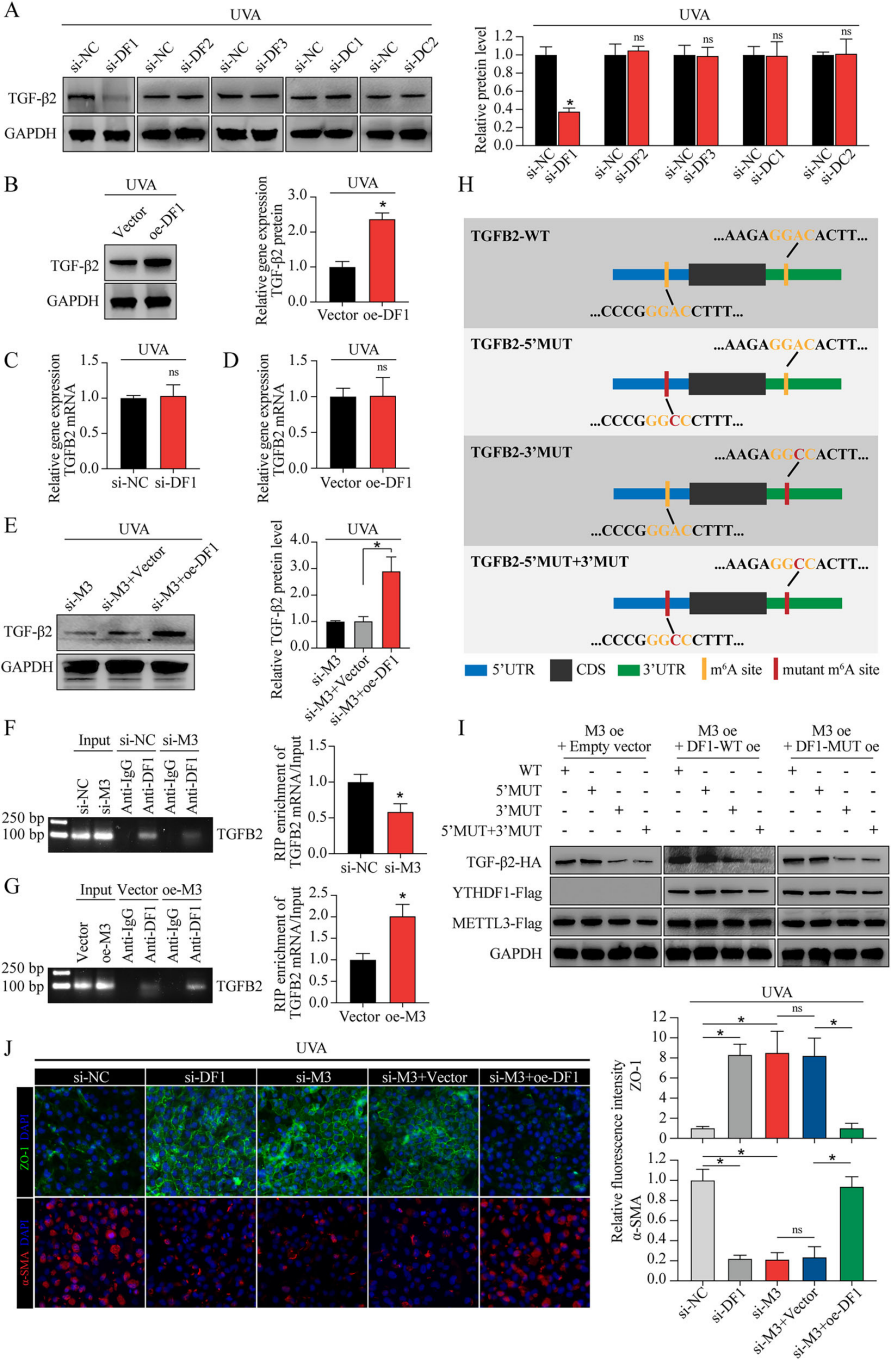
METTL3 regulates the translation of TGF-β2 in an YTHDF1-dependent manner. **A** Western blot assay of TGF-β2 protein (left panel) and relative quantitative analysis (right panel) in in vitro model with different treatments. **B** Western blot assay of TGF-β2 protein (left panel) and relative quantitative analysis (right panel) in in vitro model with or without YTHDF1 overexpression. **C** qRT-PCR analysis of *TGFB2* mRNA in in vitro model with or without YTHDF1 silencing. **D** qRT-PCR analysis of *TGFB2* mRNA in in vitro model with or without YTHDF1 overexpression. **E** Western blot assay of TGF-β2 protein (left panel) and relative quantitative analysis (right panel) in in vitro model with different treatments. RIP-qPCR using anti-YTHDF1 antibody to evaluate the binding abilities of YTHDF1 with *TGFB2* mRNA in CECs with si-M3 or si-NC treatment (**F**), as well as in CECs with oe-M3 or vector treatment (**G**). The input and anti-IgG antibody were used as positive and negative controls, respectively. Products of RT-PCR were analyzed on agarose gel and visualized with ethidium bromide staining (left panel). Enrichment of *TGFB2* mRNA was analyzed (right panel). **H** Schematic diagram of different HA-tagged *TGFB2* construction, including wild-type (*TGFB2*-WT), as well as mutations of the “GGAC” motifs in 5′UTR (*TGFB2*-5′MUT), 3′UTR (*TGFB2*-3′UTR), and both mutants (*TGFB2*-5′MUT + 3′MUT). **I** Protein level of HA-tagged *TGFB2* in CECs with different treatments. **J** Immunofluorescence staining of ZO-1 and α-SMA in in vivo model with different treatments (left panel). The fluorescence intensity was analyzed (right panel). Data are presented as means ± SD from three independent experiments. *P < 0.05, ns, not significant.

The function of m^6^A in regulating gene expression is executed mostly through recruiting m^6^A-binding proteins (readers), such as YT521-B homology (YTH) domain-containing family proteins (including YTHDF1, YTHDF2, YTHDF3, YTHDC1, and YTHDC2) in mammalian cells [35]. Due to the regulatory function of METTL3-m^6^A-TGF-β signaling axis in FECD, as well as the key role of TGF-β2 in TGF-β signaling activation, we further clarified which “reader” mainly responsible for the interaction of METTL3-m^6^A and *TGFB2* mRNA. UVA irradiation or METTL3 knockdown did not affect the levels of YTH domain-containing family proteins (Supplemental Fig. 13A, B). Knockdown of YTHDF2, YTHDF3, YTHDC1, or YTHDC2 had no effect on the protein levels of TGF-β2 (Fig. 6A). However, YTHDF1 knockdown or overexpression could mediate the protein level of TGF-β2 (Fig. 6A, B) without affecting the mRNA level of *TGFB2* (Fig. 6C, D). Forced expression of YTHDF1 also rescued the decreased TGF-β2 protein level in METTL3-silencing CECs (Fig. 6E).

To elucidate whether YTHDF1-mediated TGF-β2 expression was dependent on m^6^A modification, we first conducted RIP-qPCR. The results confirmed m^6^A modified *TGFB2* mRNA as target of YTHDF1 (Fig. 6F, G). Next, we identified “GGAC” m^6^A motifs in *TGFB2* mRNA based on our sequencing data, then constructed *TGFB2*-overexpressing plasmid with or without “GGAC” to “GGCC” mutation (Fig. 6H). Overexpression of wild-type YTHDF1 (YTHDF1-WT) led to increased TGF-β2 expression in *TGFB2*-3′WT combined with *TGFB2*-5′WT or *TGFB2*-5′MUT, while no response was observed in *TGFB2*-3′MUT combined with *TGFB2*-5′WT or *TGFB2*-5′MUT (Fig. 6I). Impairment of YTHDF1 m^6^A-binding ability (YTHDF1-MUT, schematic diagram shown in Supplemental Fig. 14) abrogated the effect of YTHDF1 in promoting *TGFB2* mRNA translation, suggesting that YTHDF1 mediated TGF-β2 expression via m^6^A modification, and the m^6^A motif in the 3′ UTR of *TGFB2* mRNA was responsible for the mediation of YTHDF1 (Fig. 6I). In addition, YTHDF1 silencing could mimic the phenotypes of METTL3 silencing in cEndMT process, while YTHDF1 overexpression could partially abolish the METTL3 silencing-alleviated cEndMT process (Fig. 6J). Collectively, these results indicate that METTL3 regulates the translation of TGF-β2 in an YTHDF1-dependent manner.

## Discussion

In the current study, we found that m^6^A “writer” methyltransferase-like 3 (METTL3) plays important roles in corneal endothelial-mesenchymal transition (cEndMT) process and Fuchs endothelial corneal dystrophy (FECD) development (shown in Graphical Abstract). METTL3 is upregulated in FECD models, leading to increased m^6^A level of *TGFB2* mRNA, upregulation of TGF-β2 protein, and activation of TGF-β signaling pathway. METTL3-m^6^A-TGF-β signaling axis mediates cEndMT process in vitro and in vivo, and subsequently promotes FECD development in vivo. At the molecular level, METTL3-m^6^A promotes the translation of TGF-β2 protein via YTHDF1 mechanism. Overall, our findings demonstrate a unique METTL3-m^6^A-mediated mechanism in regulating cEndMT process, providing a novel therapeutic strategy for FECD.

FECD used to be considered as a heterogeneous genetic disease, whereas the genetic variations do not conform to a specific clinical presentation. This points to a strong component of environmental factors involved in the onset and progression of FECD [6]. Given human corneal endothelial cells (CECs) are exposed to UV light throughout life, FECD models based on UV irradiation have been successfully established [3, 31]. Using these models, we observed aberrant m^6^A modification levels between FECD groups and normal groups through m^6^A-seq technique, suggesting the potential role of epigenetic modification in FECD. A total of 1163 differential m^6^A peaks were detected, and combined bioinformatics analyses of m^6^A-seq and RNA-seq revealed that transcripts harbouring differential m^6^A peaks were enriched in TGF-β signaling pathway. The activation of TGF-β signaling pathway in FECD was confirmed in vitro and in vivo. TGF-β signaling is a well-known regulator orchestrating both epithelial- and endothelial-mesenchymal transition [36–38]. Meanwhile, in FECD, the phenotypic switch of CECs to mesenchymal cells, cEndMT, is an important pathological process, which could explain the hallmarks detected in FECD patients, including abnormal cell morphology and excessive extracellular deposits [1].

Next, the relationship between m^6^A modification, TGF-β signaling and the cEndMT process was subjected to investigation. The role of m^6^A in TGF-β signaling has been explored by a number of researchers. To illustrate, in non-small cell lung cancer, METTL3-mediated m^6^A modification could enhance the stability of methylated lncRNA-RMRP transcripts, which further promotes *TGFBR1* transcription and the activation of TGFBR1/SMAD2/SMAD3 signaling pathway [39]. In addition, RALYL may interact with FTO in order to decrease the m^6^A modification of *TGFB2* mRNA and thereby maintain its stability. This results in the sustained secretion of TGF-β2 and the activation of PI3K/AKT/STAT3, which in turn increases the stemness of hepatocellular carcinoma [40]. Our findings indicate that the primary mechanism underlying TGF-β signaling activation in FECD is the upregulation of TGF-β2 protein level, and *TGFB2* mRNA serves as a downstream target of METTL3-mediated m^6^A modification. These outcomes are consistent with the findings of a previous study, which has demonstrated elevation of TGF-β2 protein level and activation of TGF-β signaling in FECD clinical samples [15]. Intervention of METTL3 expression (sh-M3), m^6^A catalytic activity (STM2457), or TGF-β signaling activation (LY2109761) could regulate cEndMT process in vitro and in vivo, subsequently influence FECD progression in vivo. These results revealed the role of METTL3-m^6^A-TGF-β signaling axis in cEndMT and FECD.

As the most prevalent chemical modification in mRNA of eukaryotic cells, m^6^A modification influences mRNA fate by recruiting m^6^A “readers”. The “readers” mainly refer to YTH domain–containing family proteins, including YTHDF1, YTHDF2, YTHDF3, YTHDC1, and YTHDC2 [35]. Previous studies have reported the role of METTL3-m^6^A-“reader” in phenotypic switch in different cell types. For example, in vascular smooth muscle cells, METTL3 enhance PFN1 translation efficiency via YTHDF3, then promotes phenotype switching and neointimal hyperplasia [41]. Here, we identified YTHDF1 as the “reader” binding m^6^A motif in *TGFB2* mRNA and as the modulator of *TGFB2* mRNA translation. Intervention of YTHDF1 expression could affect the role of METTL3 in TGF-β2 expression and cEndMT process. Therefore, METTL3 regulated *TGFB2* mRNA translation and cEndMT process in an YTHDF1-dependent manner.

It must be acknowledged that our research is not without limitations. Firstly, the model employed in this study is predominantly influenced by external environmental stimuli, while the development of FECD is attributed to a complex interplay between genetic and environmental factors. A novel genetic model for FECD is available using double mutant mice (*Slc4a11* and the *Col8a2*) [42]. Nevertheless, the model we employed elucidates the underlying mechanisms and regulatory molecules that target cEndMT in FECD, and is consistent with the findings of previous studies. This demonstrates the scientific value of our study. Secondly, the development of FECD is associated with a number of mechanisms, with oxidative damage representing a significant contributing factor. Our previous studies have demonstrated that long non-coding RNAs play roles in the regulation of CEC functions under oxidative stress, including proliferation, apoptosis, and anti-oxidative stress ability [43]. This study solely investigated the modulation of epigenetic modification m^6^A in cEndMT, without further exploration in oxidative damage. This does not negate the role of m^6^A in the regulation of CEC functions under oxidative stress. Thirdly, corneal edema represents one of the clinical manifestations of FECD. In the present study, we examined the effects of different interventions on corneal edema in mice. However, the results indicated the presence of inhomogeneity in corneal edema, defined as a significant variation in corneal thickness across different regions of the same cornea, which introduces greater variability in the data within the same group. Despite obtaining analogous outcomes to the METTL3 knockdown group in both the STM2457 and LY2109761 groups, these results have not been included in the text and are only illustrated with data from the METTL3 knockdown group.

In summary, we provide compelling in vitro and in vivo evidence demonstrating that METTL3 could enhance *TGFB2* mRNA translation and trigger TGF-β signaling cascades, thereby modulating cEndMT process and FECD progression. Our findings provide novel insights into the molecular mechanisms underlying FECD-related CEC dysfunction by elucidating epigenetic gene regulation. Given the functional importance of METTL3 in FECD, targeting METTL3 signaling by selective inhibitors might serve as a promising therapeutic strategy to delay FECD progression.

## Materials and methods

### Ethics statement

C57BL/6 wild-type mice (male, 8 to 12 weeks old) were used in this study. Mice were housed in a controlled temperature and humidity environment with 12-h light/dark cycle. All of the experiments and procedures were approved by the Animal Care and Use Committee of Eye & ENT Hospital (Shanghai, China) and adhered to the ARVO Statement for the Use of Animals in Ophthalmic and Vision Research.

### Cell culture

Human immortalized corneal endothelial cells (CECs, B4G12) were purchased from Creative Bioarray (Shirley, USA). CECs were seeded into plastic dishes precoated with extracellular matrix solution (Sigma-Aldrich, containing 10 mg/ml chondroitin sulfate and 10 μg/ml of laminin), then cultured in human endothelial serum-free medium (Gibco Invitrogen) supplemented with 10 ng/ml recombinant human basic fibroblast growth factor (Peprotech, UK) and 3% fetal bovine serum (FBS, Gibco Invitrogen) in 5% CO_2_ atmosphere at 37 °C. Cells were ready for passaging at 80–90% confluence.

### Establishment of UVA-induced FECD models

For in vitro FECD model establishment, a UVA lamp emitting 365 nm light (UVL-26; UVP, Upland, CA, USA) was applied to irradiate normal CECs at a fluence of 5 J/cm^2^ (irradiance: 5 mW/cm^2^; exposure time: 1000 s). UVA-treated cells were incubated in 5% CO_2_ atmosphere at 37 °C, and collected 4 days after UVA exposure. For in vivo FECD model establishment, mice were anesthetized using ketamine/xylazine (100 mg/kg ketamine and 20 mg/kg xylazine) intraperitoneally. A UVA LED light source emitting 365 nm light (M365LP1-C1; Thorlabs) was applied to illuminate a 4-mm-diameter spot onto the central cornea using a convex lens (LB4972-uv; Thorlabs). The time of UVA exposure was adjusted to deliver a fluence of 150 J/cm^2^ (irradiance: 120 mW/cm^2^; exposure time: 1250 s). The other untreated eye was served as a control. To guarantee an adequate sample size, more than 25 mice were irradiated at a time, then randomly divided into 5 groups, with the objective of ensuring that there were four to five mice in each group at the time of sample collection. The group assignment, anterior chamber injection, and assessment of CECs were conducted in a double-blind manner. UVA-treated corneas were collected 14 days after UVA exposure.

### Cell transfection

CECs were transfected with synthesized small interfering RNAs (siRNAs) or plasmids purchased from YiXueSheng Biosciences Inc (Shanghai, China) and RiboBio Company (Guangzhou, China) using transfection reagent in accordance with the manufacturer’s instructions. The sequences of siRNAs were listed in Supplemental Table 1.

### Anterior chamber injection

Mice were anesthetized using ketamine/xylazine (100 mg/kg ketamine and 20 mg/kg xylazine) intraperitoneally. One drop of tropicamide was used to dilate the pupil, and lubricant eye gel was applied to protect the cornea. After full anesthesia and pupil dilation, a 30-gauge beveled needle was inserted into the anterior chamber (about 1.5 mm depth) at 1 mm anterior to the corneal limbus with a 45° angle under the microscope, then gently removed. About 2 μl preload solution was slowly (over a course of 1 min) injected into the anterior chamber using a Hamilton microsyringe fitted to a 33-gauge needle. After complete injection, the needle was held in place for an additional minute, then gently removed. Antibiotic ointment was applied to the needle wound to prevent infection. The adeno-associated virus (AAV) targeting mouse *Mettl3* was designed and synthesized by Shandong Weizhen Company (Jinan, China). STM2457 (Cat.No. HY-134836) and LY2109761 (Cat.No. HY-12075) were purchased from MedChemExpress (Shanghai, China).

### RNA m^6^A dot blot assay

RNA m^6^A dot blot assay was conducted using total RNA. The samples were adjusted to the concentration of 500 ng RNA/μl or 250 ng RNA/μl, and a total of 2 μl samples were loaded onto a nitrocellulose membrane (FFN02, Beyotime). RNA on the membrane was UV cross-linked for 5 min, blocked with 5% skim milk for 1 h, incubated with m^6^A-specific antibody (1:1000, ab208577, Abcam) for 2 h at room temperature (RT), and then incubated with HRP-conjugate anti-mouse immunoglobulin G (1:1000, 115-035-003, Jackson ImmunoResearch) for 2 h at RT. The dot blot signals were visualized using imaging system (JS-1070P, Peiqing Science & Technology) after incubation with ECL reagent (RPN2232, GE Healthcare). Methylene blue (0.02%, M9140, Sigma-Aldrich) in 0.3 M sodium acetate (pH 5.2) was employed to confirm equal RNA loading. The signal density of each dot was quantified using ImageJ software.

### m^6^A-sequencing (m^6^A-seq) and RNA-sequencing (RNA-seq)

Total RNA from CEC lysate was isolated using Trizol reagent (Invitrogen, CA, USA). RNA integrity was tested using Agilent 2100 Bioanalyzer (Agilent Technologies, Santa Clara, CA, USA), and samples with RNA Integrity Number > 7.0 were subjected to further study. RNA samples were fragmented into 150-nucleotide-long oligonucleotides using divalent cations and thermocycler at 94 °C for exactly 5 min, then divided into two portions. One portion was saved as input fragments to construct RNA-seq library, while the other portion was saved as RIP fragments (m^6^A-positive RNA fragments) to construct m^6^A-seq library. In brief, cleaved RNA fragments were incubated with m^6^A-specific antibody (No.202003, Synaptic Systems, Germany) for 2 h at 4 °C. The mixture was incubated with protein-A beads, eluted with elution buffer, and precipitated using 75% ethanol to obtain RIP fragments. Thereafter, input fragments and RIP fragments were converted to final RNA-seq library and m^6^A-seq library using dUTP method in according to our previous study [43]. The average insert size for the paired-end libraries was ~150 bp. The libraries were sequenced on the Illumina sequencing platform (NovaSeq 6000) following the vendor’s recommended protocol.

### Bioinformatics analysis

m^6^A peaks were identified using MeTDiff peak calling software, and m^6^A peak distribution was visualized using Integrative Genomics Viewer (IGV) software. Differentially methylated peaks between two groups were determined using MeTDiff with a threshold of foldchange (FC) ≥ 1.5 and *P* value < 0.05. Sequence motifs were detected using MEME and DREME, then annotated using Tomtom software. Transcripts with FC ≥ 1.5 and *P* value < 0.05 were described as differentially expressed between two groups. Gene ontology (GO) analysis and Kyoto Encyclopedia of Genes and Genomes (KEGG) pathway analysis of transcripts harbouring differential peaks were conducted using R based on hypergeometric distribution. Gene set enrichment analysis (GSEA) was conducted using GSEA R package.

### Western blot (WB) analysis

Quantified protein samples were resolved on sodium dodecyl sulfate–polyacrylamide gel electrophoresis (SDS-PAGE) and transferred to a polyvinylidene fluoride (PVDF) membrane. The membrane was blocked with 5% non-fat milk in Tris-buffered saline-Tween 20 (TBS-T) for 1 h at RT, incubated with primary antibody in 5% bovine serum albumin (BSA) in TBS-T overnight at 4 °C, and then incubated with horseradish peroxidase (HRP)-conjugated secondary antibody for 1 h at RT. The membrane was visualized using ECL reagent (RPN2232, GE Healthcare). The band signals were detected using Fluorescent & Chemiluminescence Gel Imaging System (JS-1070P, Peiqing Science & Technology). Antibodies used in this study were listed in Supplemental Table 2.

### Immunofluorescence (IF) staining

Cells were fixed with 75% ethanol for 20 min at RT, permeabilized with 0.3% Triton for 10 min at RT, and then blocked using 5% FBS for 1 h at 37 °C. Corneal tissues were isolated from eyeballs, fixed with 4% paraformaldehyde (PFA) for 1 h at RT, permeabilized with 0.3% Triton for 30 min at RT, and then blocked using 3% BSA for 30 min at 37 °C. Cells or tissues were incubated with primary antibody in phosphate buffer saline (PBS) overnight at 4 °C, and then incubated with secondary antibody in PBS for 1 h at RT. 4,6-diamidino-2-phenylindole (DAPI; D9542; Sigma-Aldrich) was employed for nucleic acids staining. After addition of anti-fluorescence quenching agent, cells were visualized under fluorescence microscope (Olympus IX-73, Tokyo, Japan), while central corneal tissues were visualized under confocal microscope (Leica SP8; Wetzlar, Germany). Antibodies used in this study were listed in Supplemental Table 2.

### Quantitative reverse transcription PCR (qRT-PCR) analysis

Total RNA from cells or tissues was extracted using RNAsimple Total RNA Kit (Tiangen, Beijing, China). Complementary DNA (cDNA) was synthesized using Fastking gDNA Dispelling RT SuperMix (Tiangen, Beijing, China) according to the manufacturer’s instructions. Quantitative PCR was performed using SYBR Green PCR Kit (Qiagen, Hilden, Germany). All qRT-PCR reactions were tested in triplicates. Relative gene expression was normalized to the expression of glyceraldehyde 3-phosphate dehydrogenase (GAPDH) as a reference gene. Primers used in this study were listed in Supplemental Table 3.

### m^6^A-modifed RNA immunoprecipitation combined with qRT-PCR (MeRIP-qPCR)

MeRIP was performed using the PureBinding®RNA Immunoprecipitation Kit (Cat.No. P0102, Geneseed, China) following the manufacturer’s instructions. m^6^A enrichment was detected using qRT-PCR analysis as described above.

### Evaluation of FECD progression

Confocal microscopy (CM) was used to help evaluate the density, size, and morphology of CECs located in central cornea. Slit lamp biomicroscope and optical coherence tomography were employed to assess the degree of corneal edema. Optical coherence tomography (OCT) was used to detected the thickness of corneas. All these procedures were performed with the assistance of clinical technicians. Confocal ZO-1 immunostaining images at 40X magnification were used for analysis of density, size, and pleomorphism. Briefly, a circular range of the central cornea (r = 125 μm) was selected. The number of nuclei within the circle were counted, and cell density was calculated using the cell number divided by the circular area (cells/mm^2^). The average cell size was calculated using the circular area divided by the cell number (mm^2^). Pleomorphism was assessed by calculating the proportion of non-hexagonal cells (%). The schematic diagram of the calculation refers to the Supplemental Fig. 12.

### Statistical analyses

Data are presented as the mean ± standard deviation (SD) of at least three independent experiments. Student’s *t*-test or one-way ANOVA was conducted using the GraphPad Prism software. Values of *P* < 0.05 was considered to be statistically significant.

## Supplementary information

supplemental information

supplementary file WB

## Data Availability

The datasets used and/or analysed during the current study are available from the corresponding author on reasonable request.
