# Supplementary material for: METTL3-m6A-mediated TGF-β signaling promotes Fuchs endothelial corneal dystrophy via regulating corneal endothelial-to-mesenchymal transition
Source: Cell Death Discov. 2025 Mar 15;11:104. doi: 10.1038/s41420-025-02384-1 (PMC11910554; doi:10.1038/s41420-025-02384-1)
Supplement: Supplementary file 1 — supplemental information [file 41420_2025_2384_MOESM1_ESM.docx]

**Supplemental Information**

**Supplemental Figures: page 2-16**

**Supplemental Tables: page 17-19**

**Supplemental Materials and Methods: page 20-21**

**Supplemental Figures**


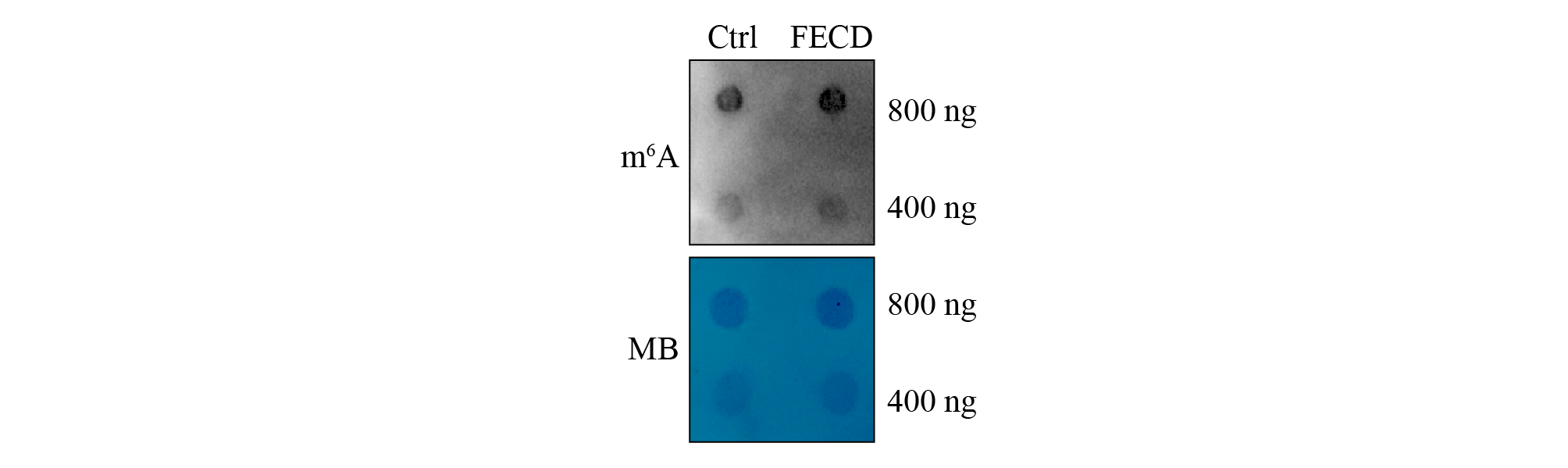


**Supplemental Figure 1.** **Total m^6^A levels in control and** **FECD groups**

Increased total m^6^A levels in FECD groups compared to control groups as shown by RNA dot blot assay.

**
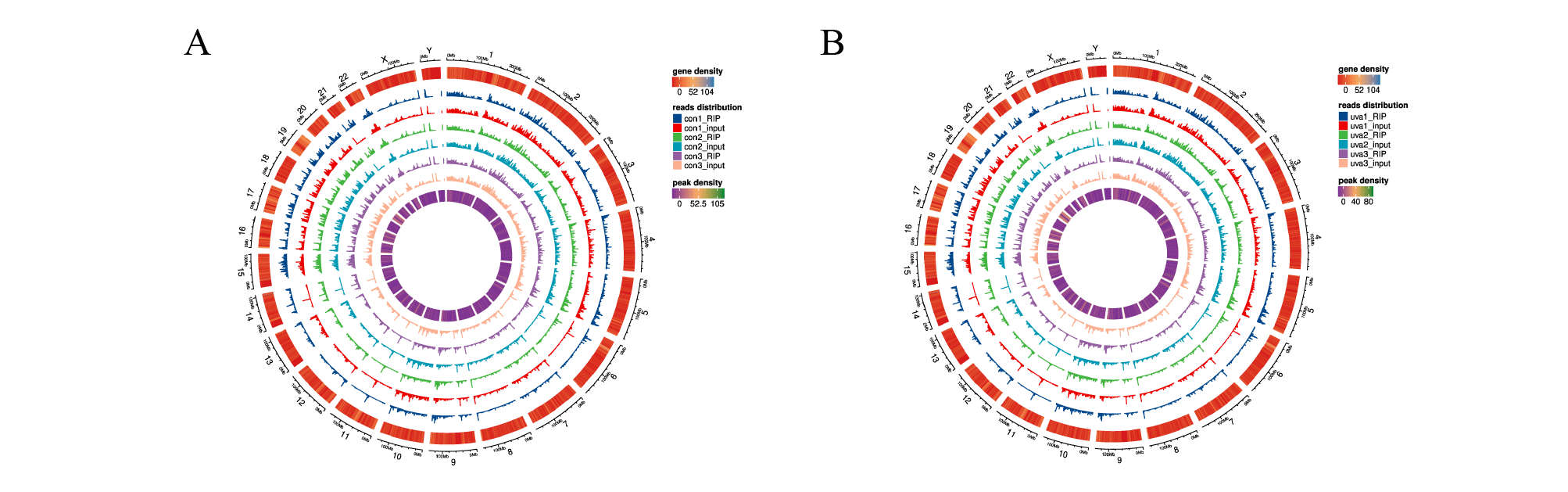
**

**Supplemental Figure 2. Distribution of m^6^A reads in human genome**

(A and B) The m^6^A reads from input and RNA immunoprecipitation (RIP) samples in control groups (A) and FECD groups (B) were aligned to human reference genome GRCh38 and visualized using "circlize" package in R software.

**
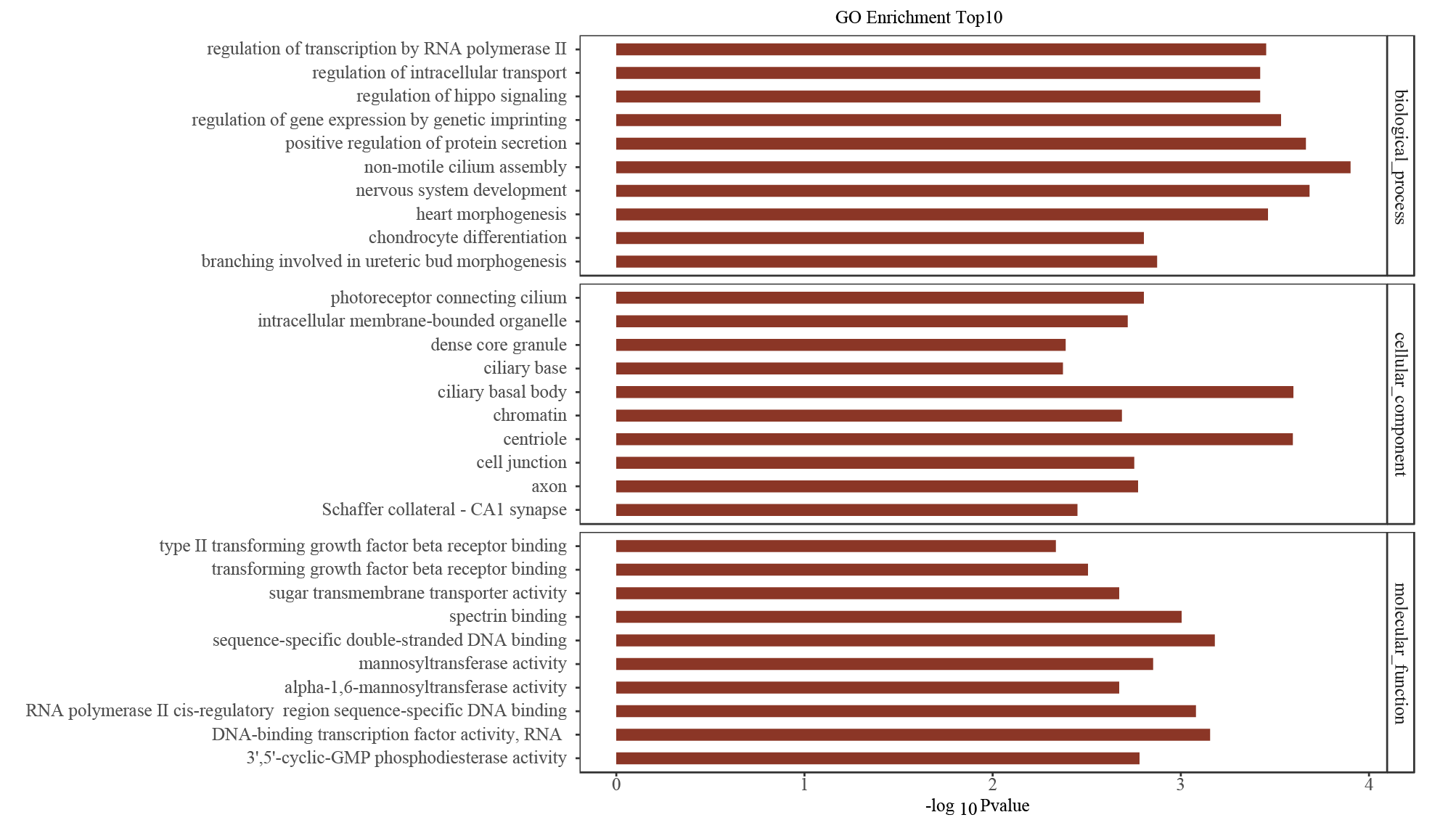
**

**Supplemental Figure 3.** Gene ontology (GO) analysis

GO analysis of transcripts harbouring differential m^6^A peaks, covering biological pro- cess, cellular component, and molecular function. The top 10 GO terms of each domain are listed.

**
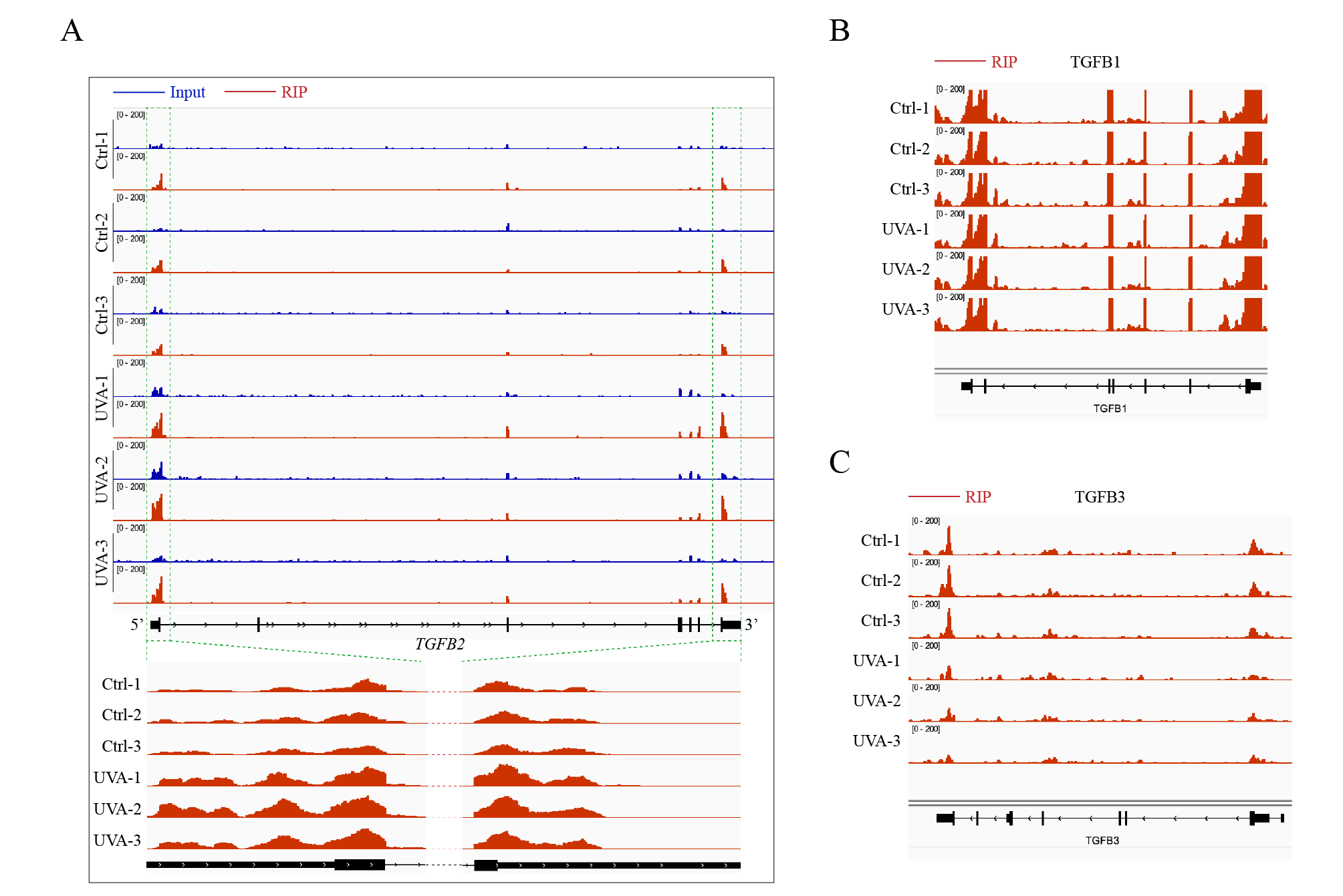
**

**Supplemental Figure 4.** **Distributions of m^6^A peaks in** **TGFB family**

(A-C) Distributions of m^6^A peaks in *TGFB2* mRNA (A), *TGFB1* mRNA (B), and *TGFB3* mRNA (C) are visualized using Integrative Genomics Viewer (IGV) software. *TGFB2* mRNA shows increased m^6^A peaks abundance in FECD groups (A). *TGFB1* mRNA shows no significant change in m^6^A peak abundance between two groups (B). *TGFB3* mRNA shows decreased m^6^A peak abundance in FECD groups (C).

**
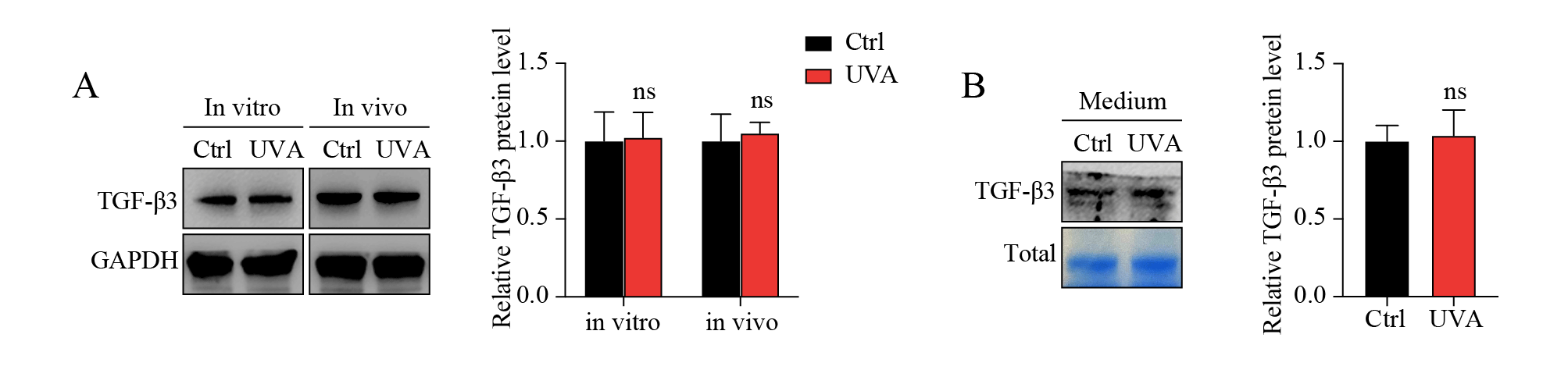
**

**Supplemental Figure 5. Detection of TGF-β3 protein level**

(A) Western blot assay of TGF-β3 expression *in vitro* and *in vivo*. Relative quantitative expression of TGF-β3 at the protein level is shown in the right panel (ns, not significant). (B) Western blot assay and relative quantitative analysis of TGF-β3 expression in cell culture medium, with Coomassie Brilliant Blue as the loading control (ns, not significant).

**
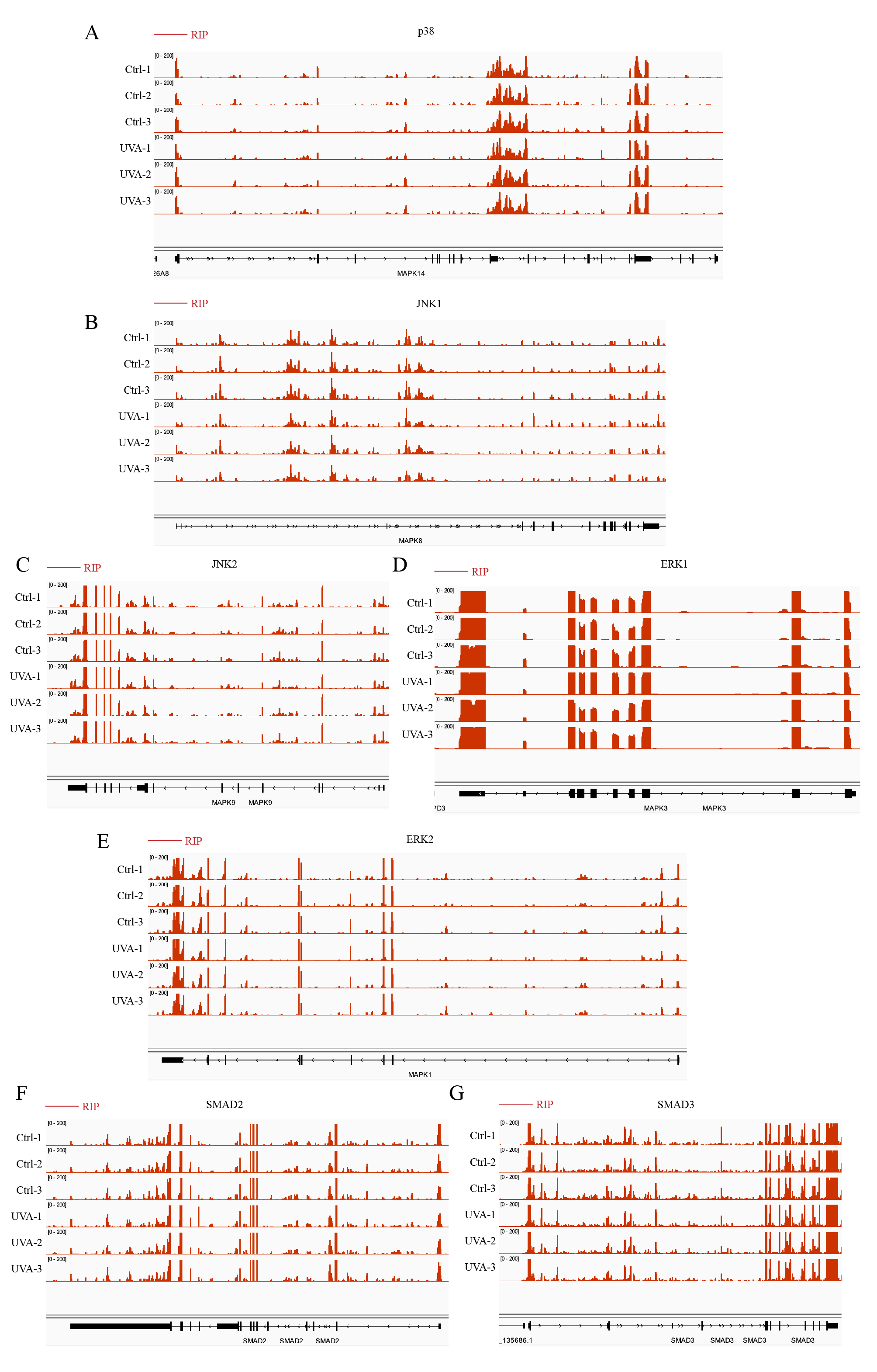
**

**Supplemental Figure 6. Distributions of m^6^A peaks in TGF-β signaling related downstream** **mediators**

(A-G) Distributions of m^6^A peaks in *p38*, *JNK1*, *JNK2*, *ERK1*, *ERK2*, *SMAD2*, and *SMAD3* mRNA are visualized using IGV software. No significant change in m^6^A peak abundance is detected.

**
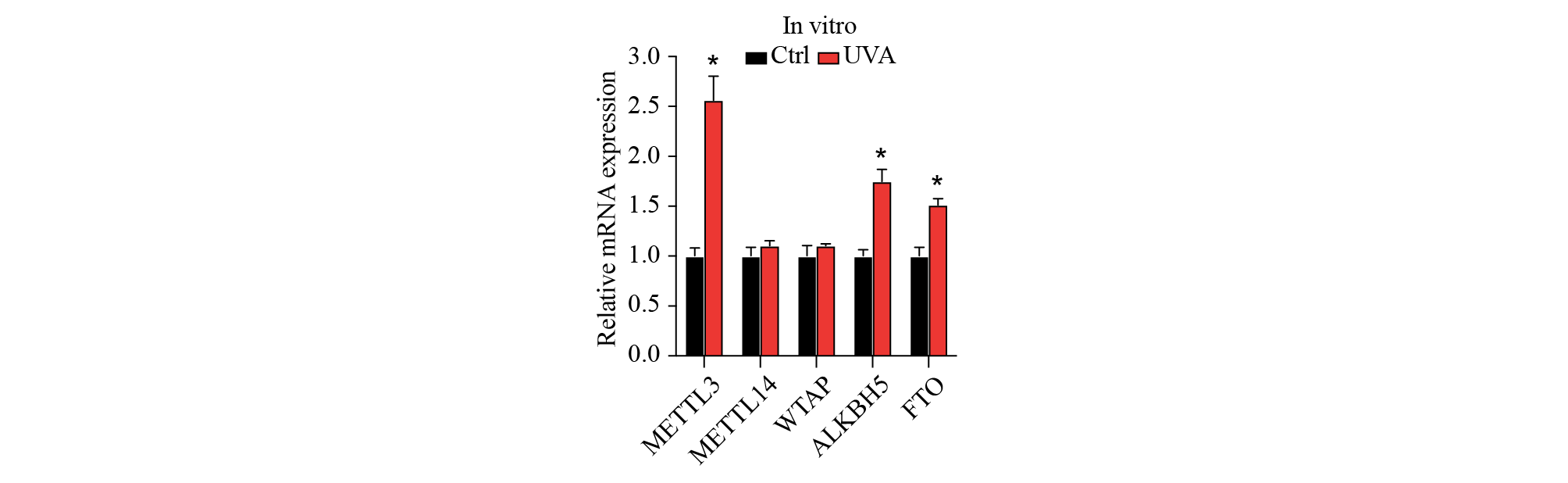
**

**Supplemental Figure 7.** **Alternation of gene expression at mRNA level** **in *in vitro* model**

Relative expression of METTL3, METTL14, WTAP, ALKBH5, and FTO at mRNA level in FECD groups compared to control groups using quantitative reverse transcription PCR (qRT-PCR) (**P* < 0.05).

**
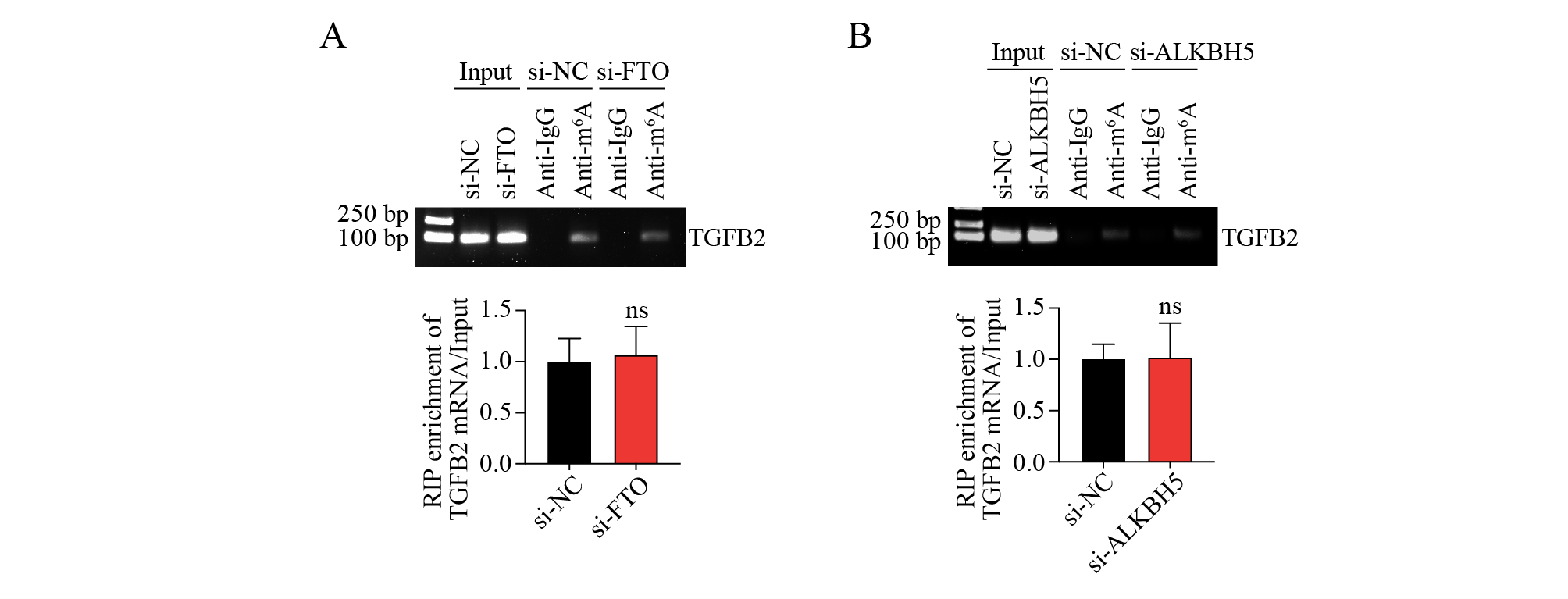
**

**Supplemental Figure 8. Evaluation of m^6^A levels in *TGFB2* mRNA**

(A and B) RIP combined with qRT-PCR analysis (RIP-qPCR) using anti-m^6^A antibody to evaluate the m^6^A levels of *TGFB2* mRNA in corneal endothelial cells (CECs) with FTO-silencing (si-FTO) or non-silencing (si-NC) treatment (A), as well as in CECs with ALKBH5-silencing (si-ALKBH5) or si-NC treatment (B). The input and anti-IgG antibody were used as positive and negative controls, respectively. Products of RT-PCR were analyzed on agarose gel and visualized with ethidium bromide staining (left panel). Enrichment of *TGFB2* mRNA was analyzed (right panel, ns, not significant).

**
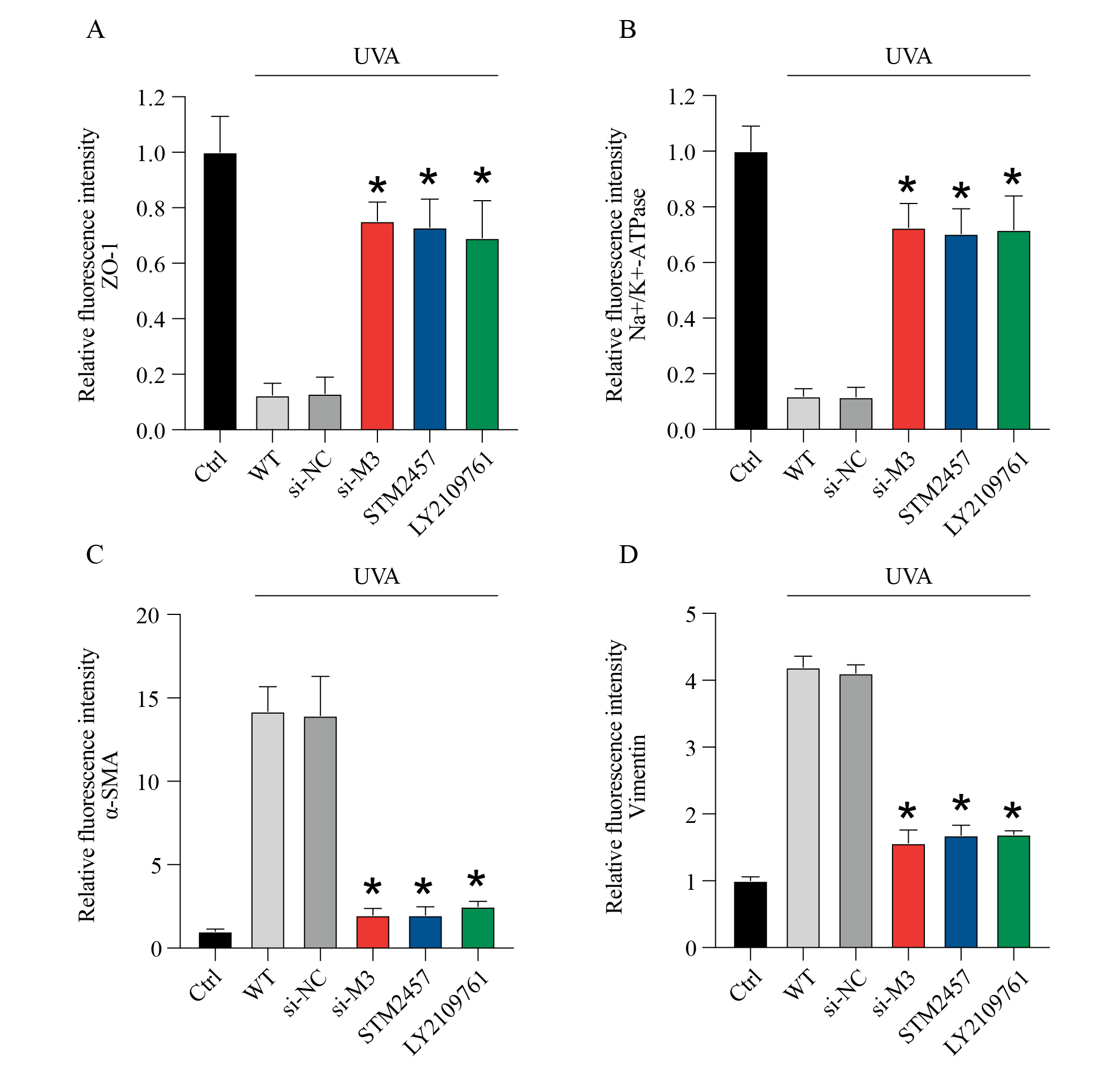
**

**Supplemental Figure 9.** **Relative fluorescence intensity of markers *in vitro***

(A-D) Relative fluorescence intensity of ZO-1, Na^+^/K^+^-ATPase, α-SMA, and vimentin *in vitro* (* *P* < 0.05 versus si-NC groups).

**
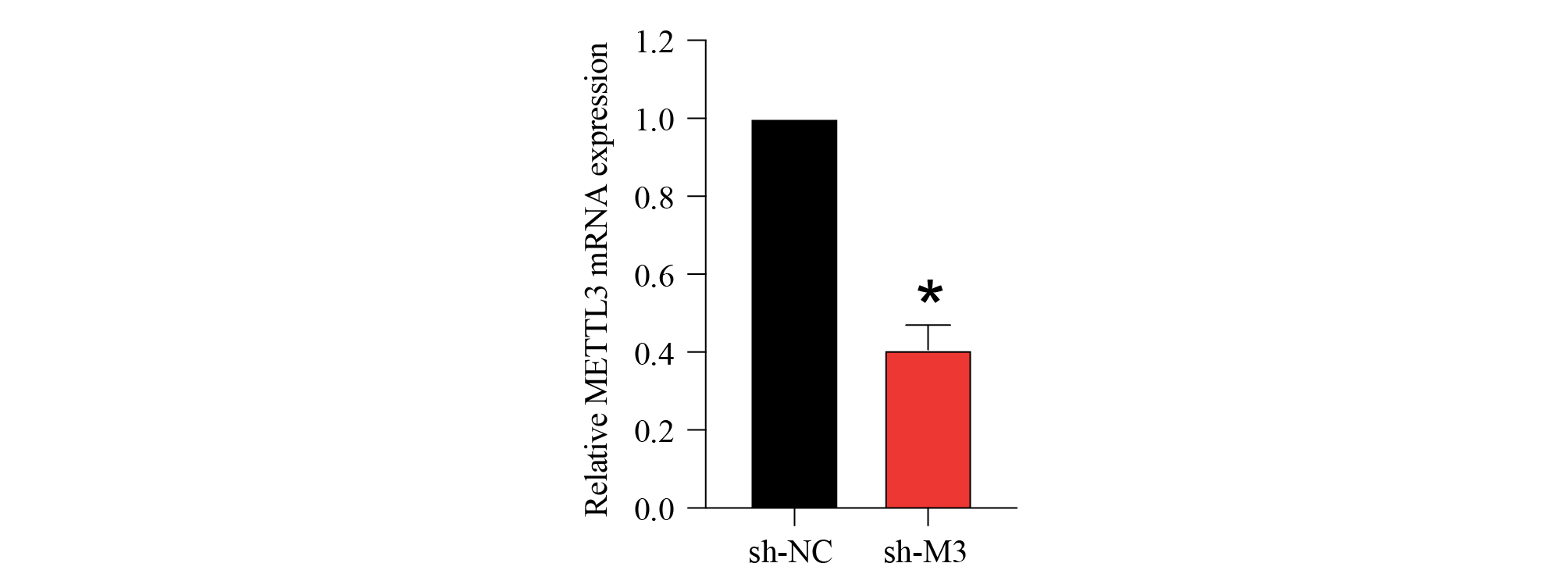
**

**Supplemental Figure 10. Silencing efficacy of AAV targeting METTL3**

Evaluation of silencing efficacy of AAV targeting METTL3 using qRT-PCR (**P* < 0.05).

**
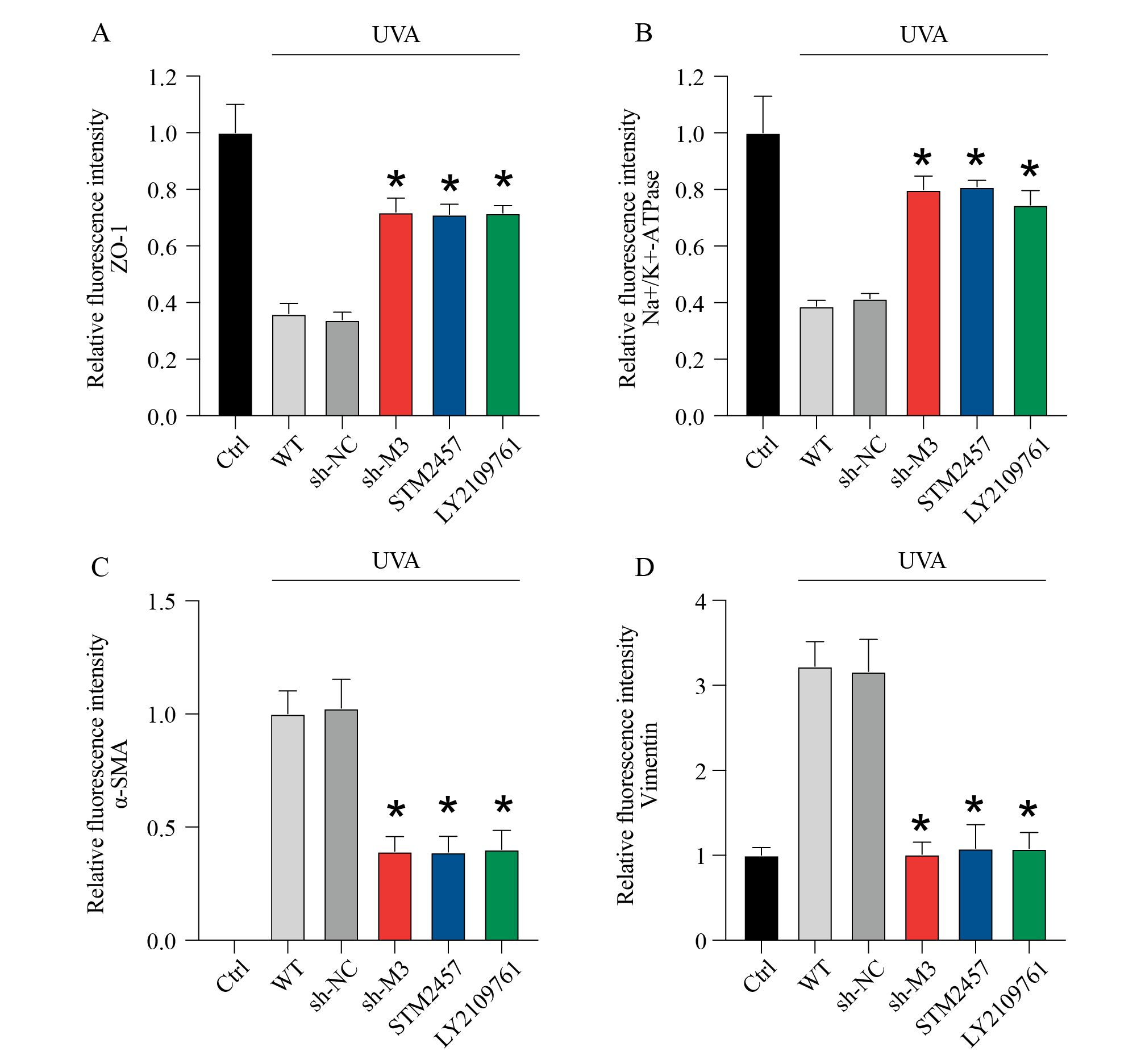
**

**Supplemental Figure 11. Relative fluorescence intensity of markers *in vivo***

(A-D) Relative fluorescence intensity of ZO-1, Na^+^/K^+^-ATPase, α-SMA, and vimentin *in vivo* (* *P* < 0.05 versus sh-NC groups).

**
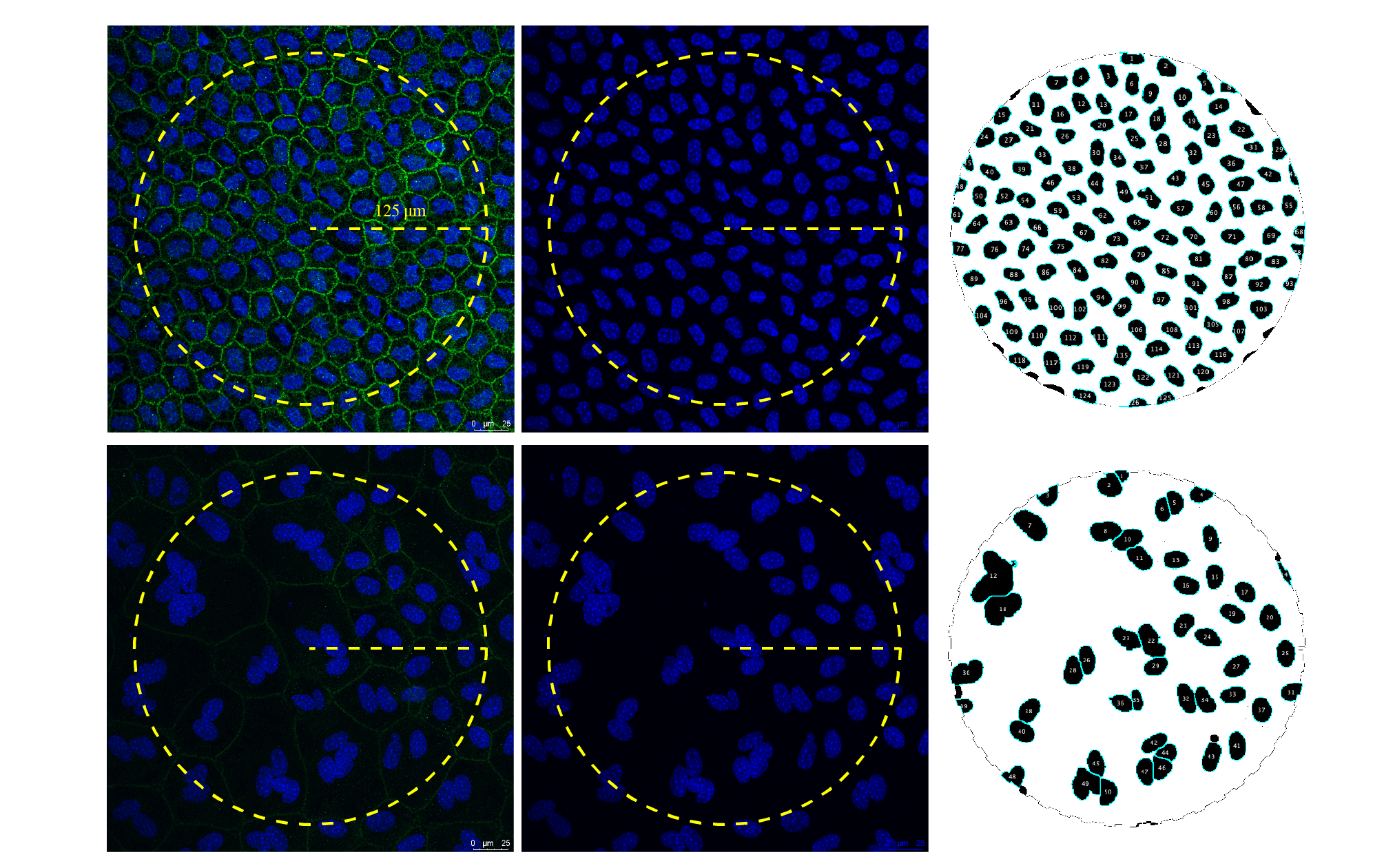
**

**Supplemental Figure 12. Schematic diagram of calculating cell density and cell size.**

Cell density and cell size of CECs were calculated as described in the schematic diagram using ImageJ.

**
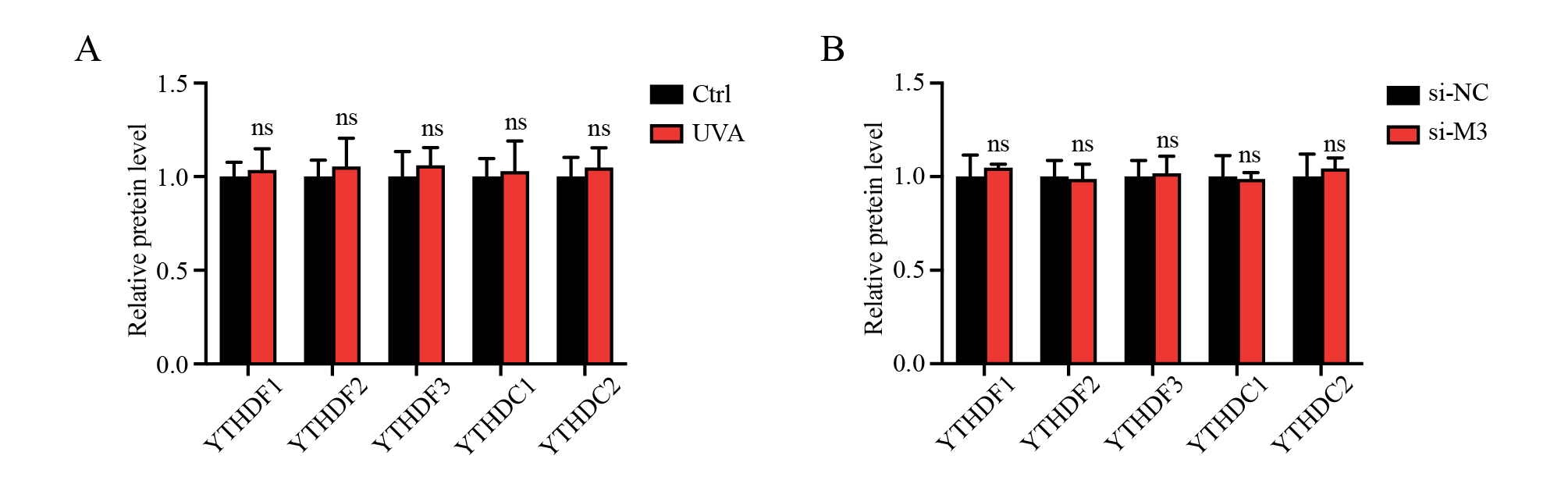
**

**Supplemental Figure 13. Detection the protein levels of YTH domain-containing family**

(A and B) Western blot assay of YTH domain-containing family proteins. UVA irradiation (A) or METTL3 silencing (B) had no influence on the expression of these proteins *in vitro* (ns, not significant).

**
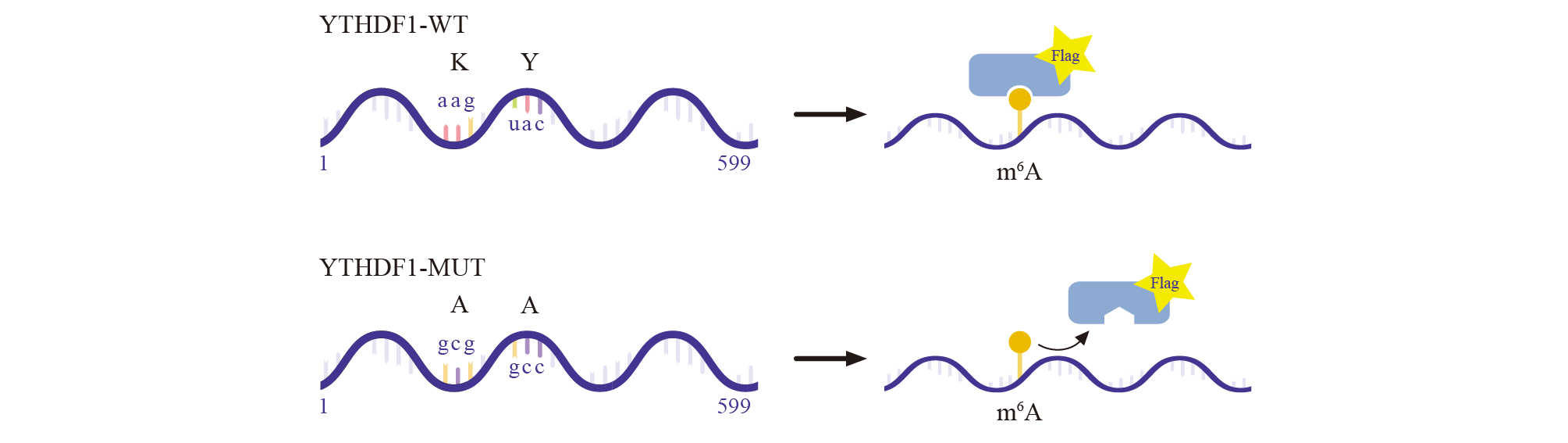
**

**Supplemental Figure 14. Schematic diagram of wild-type and mutant YTHDF1 construction.**

Schematic diagram of wild-type (YTHDF1-WT) and mutant YTHDF1 (YTHDF1-MUT) construction.

**Supplemental Tables**

**Supplemental Table 1. Sequence of siRNA and shRNA**

| si-METTL3 | sense (5’-3’) | GCUGCACUUCAGACGAAUUTT |
| --- | --- | --- |
|  | antisense (5’-3’) | AAUUCGUCUGAAGUGCAGCTT |
| si-YTHDF1 | sense (5’-3’) | GUUCGUUACAUCAGAAGGAUATT |
|  | antisense (5’-3’) | UAUCCUUCUGAUGUAACGAACTT |
| si-YTHDF2 | sense (5’-3’) | AAGGACGUUCCCAAUAGCCAATT |
|  | antisense (5’-3’) | UUGGCUAUUGGGAACGUCCUUTT |
| si-YTHDF3 | sense (5’-3’) | GAUAAGUGGAAGGGCAAAUUUTT |
|  | antisense (5’-3’) | AAAUUUGCCCUUCCACUUAUCTT |
| si-YTHDC1 | sense (5’-3’) | UGCCUCCAGAGAACCUUAUAATT |
|  | antisense (5’-3’) | UUAUAAGGUUCUCUGGAGGCATT |
| si-YTHDC2 | sense (5’-3’) | GCCUUGGAUGUAAAUCUCUUUTT |
|  | antisense (5’-3’) | AAAGAGAUUUACAUCCAAGGCTT |
| si-FTO | sense (5’-3’) | UCACCAAGGAGACUGCUAUUUTT |
|  | antisense (5’-3’) | AAAUAGCAGUCUCCUUGGUGATT |
| si-ALKBH5 | sense (5’-3’) | GCUUCAGCUCUGAGAACUATT |
|  | antisense (5’-3’) | UAGUUCUCAGAGCUGAAGCTT |
| sh-Mettl3 | 5’-GGACCAAGGAAGAGTGCATGATTCAAG  AGATCATGCACTCTTCCTTGGTCCTTTTTT-3‘ | |

**Supplemental Table 2. Antibodies used in this study.**

| Antibody | Dilution | Company |
| --- | --- | --- |
| METTL3 | 1:1000 (WB and IF) | Abcam, ab195352 |
| m^6^A | 1:1000 (Dot Blot) | ab208577 |
| TGF-β2 | 1:500 (WB) | Abbkine, ABP52599 |
| p-SMAD2/3 | 1:1000 (WB) | Cst, 18338 |
| SMAD2/3 | 1:1000 (WB) | Cst, 8685 |
| p-p38 | 1:1000 (WB) | Cst, 4511 |
| p38 | 1:1000 (WB) | Cst, 8690 |
| p-JNK1/2 | 1:1000 (WB) | Cst, 4668 |
| JNK1/2 | 1:1000 (WB) | Cst, 9252 |
| p-ERK1/2 | 1:1000 (WB) | Cst, 4370 |
| ERK1/2 | 1:1000 (WB) | Cst, 4695 |
| GAPDH | 1:5000 (WB) | Abcam, ab8245 |
| METTL14 | 1:1000 (WB) | Abcam, ab309096 |
| WTAP | 1:1000 (WB) | Abcam, ab195380 |
| ALKBH5 | 1:1000 (WB) | Abcam, ab195377 |
| FTO | 1:10000 (WB) | Abcam, ab126605 |
| ZO-1 | 1:200-1:1000 (WB)  1:100 (IF) | Invitrogen, 40-2200 |
| Na^+^/K^+^-ATPase | 1:500 (IF) | Abcam, ab76020 |
| α-SMA | 1:10000-1:50000 (WB),  1:250-1:500 (IF) | Abcam, ab124964 |
| Vimentin | 1:250-1:1000 (IF) | Abcam, ab92547 |

**Supplemental Table 3. Primers used in this study.**

| TGFB2 mRNA (human) | F | 5'-CAAGGAGGTTTACAAAATAGACAT-3' |
| --- | --- | --- |
|  | R | 5'-GTAGAAAGTGGGCGGGAT-3' |
| METTL3 mRNA (mouse) | F | 5'-GTCAACGAAAGAACAGCAGAGC-3' |
|  | R | 5'-CTTCCTTGGTCCCATAATCAC-3' |
| METTL3 mRNA (human) | F | 5'-CTATCTCCTGGCACTCGCAAGA-3' |
|  | R | 5'-GCTTGAACCGTGCAACCACATC-3' |
| METTL14 mRNA (human) | F | 5'-GTTGGAACATGGATAGCCGC-3' |
|  | R | 5'-CAATGCTGTCGGCACTTTCA-3' |
| WTAP mRNA (human) | F | 5'-CCAGCTATGCTTCAGATCGCCT-3' |
|  | R | 5'-GGTTCTCTTCCTTGTCCATCTCC-3' |
| ALKBH5 mRNA (human) | F | 5'-TTCCCAAGAAGGTTCGATTG-3' |
|  | R | 5'-TGCAGACTCCTGCTGTTGTT-3' |
| FTO mRNA (human) | F | 5'-CCAGAACCTGAGGAGAGAATGG-3' |
|  | R | 5'-CGATGTCTGTGAGGTCAAACGG-3' |

**Supplemental Materials and Methods**

**Plasmid Construction**

All plasmids were synthesized by YiXueSheng Biosciences Inc (Shanghai, China), and experiments were conducted in accordance with their methodology. The plasmids for *TGFB2* variants (WT, 5’MUT, 3’MUT, and 5’MUT+3’MUT), METTL3, YTHDF1-WT, and YTHDF1-MUT (K395A/Y397A) were constructed using the pLVX-EF1a-mNeonGreen-Puro-CMV-MCS-3Flag vector. The target sequences of *TGFB2* variants were inserted into the vector, including a C-terminal HA tag followed by a stop codon. The METTL3 sequence was inserted into the vector, with a Kozak sequence (GCCACC) added before the ATG start codon. The YTHDF1 sequence was inserted into the vector, with a Kozak sequence (GCCACC) added before the ATG start codon and a 3xFlag tag added to the C-terminus. The YTHDF1 sequence was inserted into the vector, with specific mutations introduced at positions 395 (K395A, AAG to GCG) and 397 (Y397A, TAC to GCC). A Kozak sequence (GCCACC) was added before the ATG start codon, and a 3xFlag tag was added to the C-terminus. The EF1a promoter drives the expression of mNeonGreen, while the CMV promoter drives the expression of the target gene. mNeonGreen and the target gene are expressed independently without fusion.

All constructed plasmids were subjected to Sanger sequencing to ensure the correct insertion of the target sequences, the presence of the Kozak sequence (where applicable), and the correct incorporation of protein tags and mutations. Sequencing results were analyzed using appropriate bioinformatics tools to confirm the accuracy of the constructs.

**STM2457 and LY2109761 application *in vitro***

STM2457 and LY2109761 were both administered subsequent to UV irradiation at a concentration of 1 μM in accordance with preceding studies (PMID 33902106 and 30355908).
