# Supplementary figures and images for: METTL3-m6A-mediated TGF-β signaling promotes Fuchs endothelial corneal dystrophy via regulating corneal endothelial-to-mesenchymal transition
Source: Cell Death Discov. 2025 Mar 15;11:104. doi: 10.1038/s41420-025-02384-1 (PMC11910554; doi:10.1038/s41420-025-02384-1)

Figure 2

C

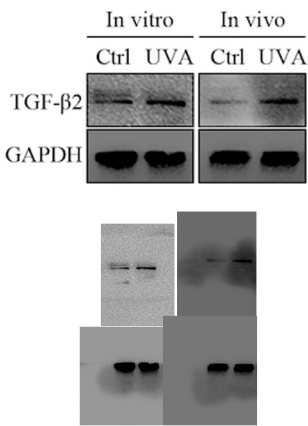

D

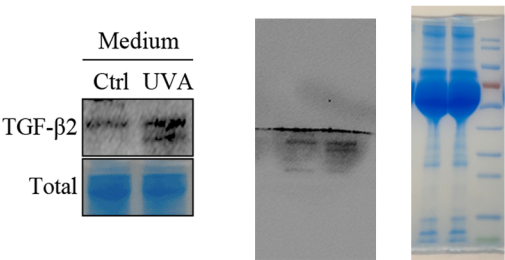

E

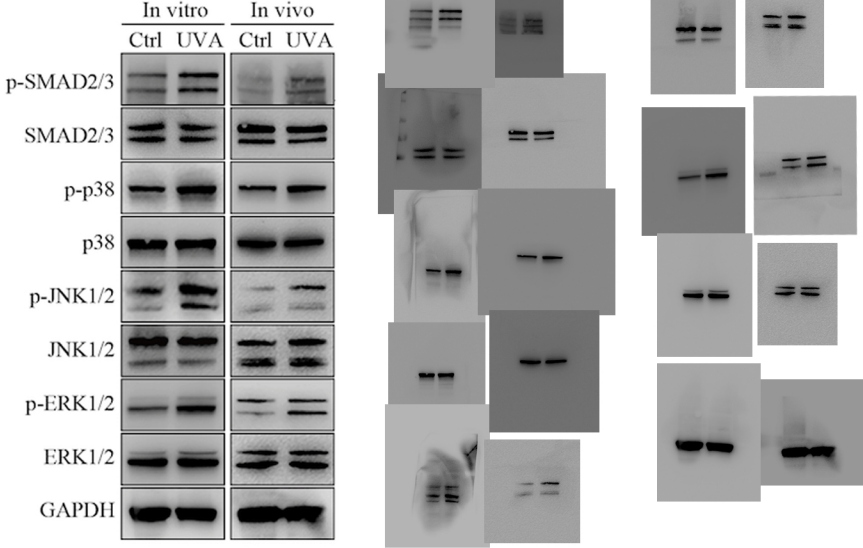

Figure 3

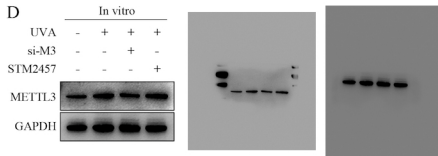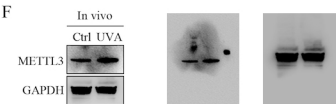

H

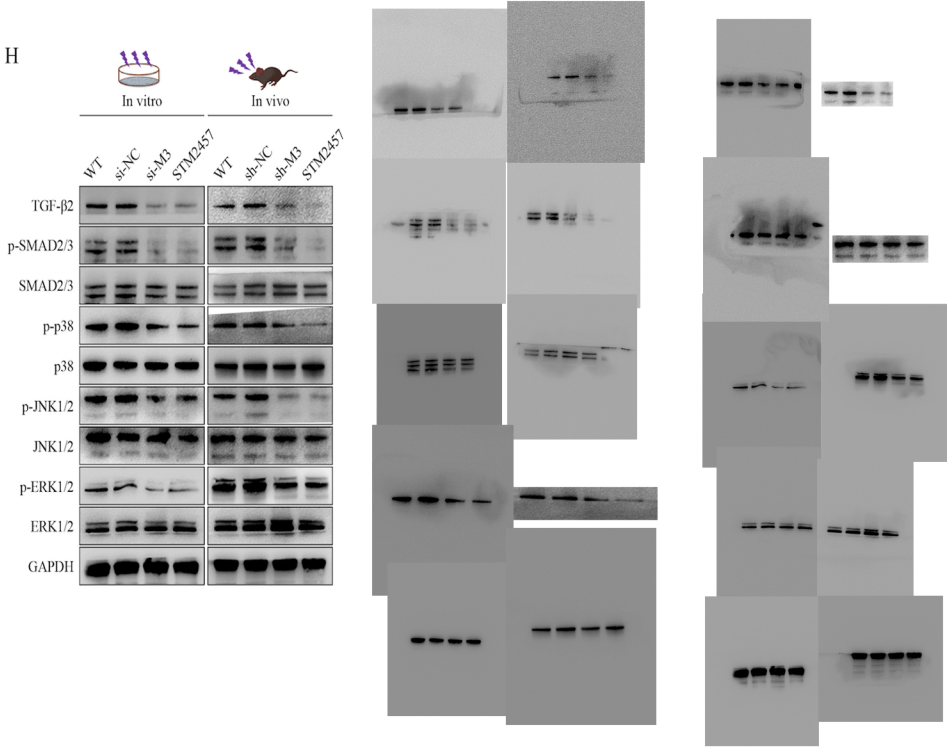

Figure 4

Figure 5

C

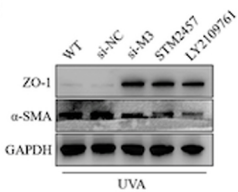

B

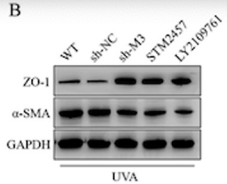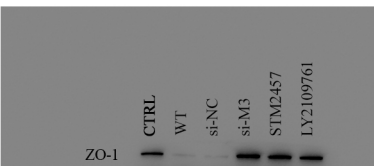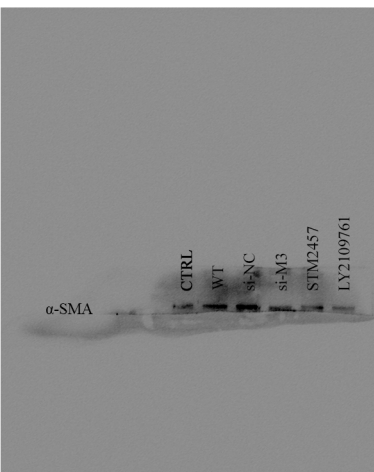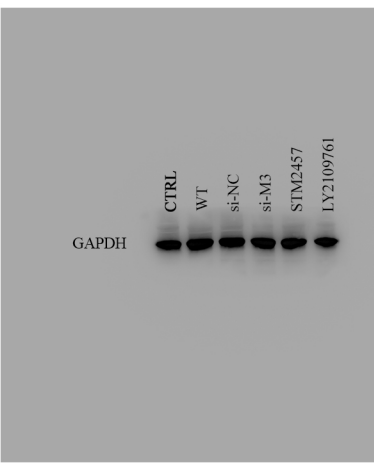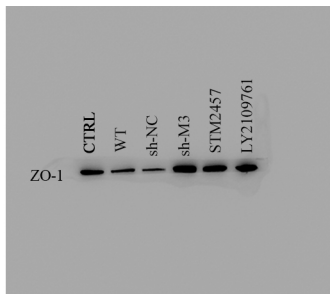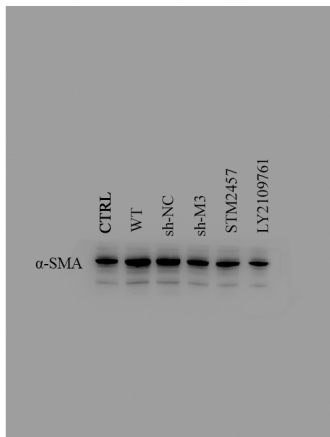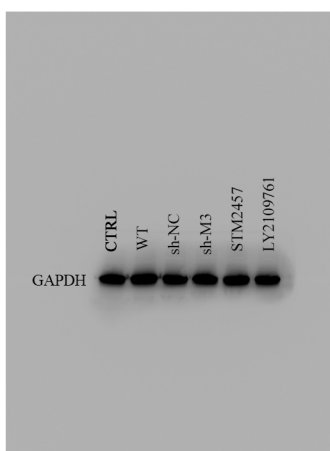

Figure 6

E

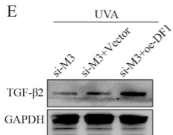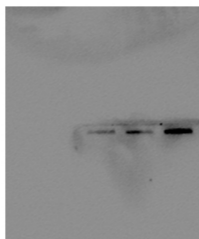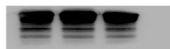

I

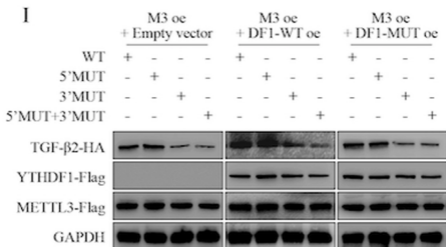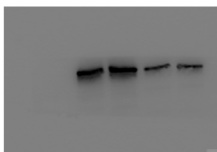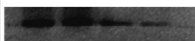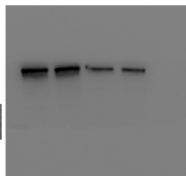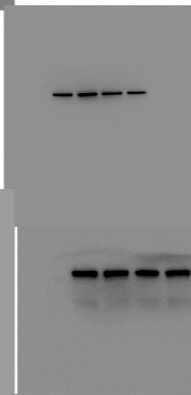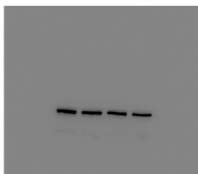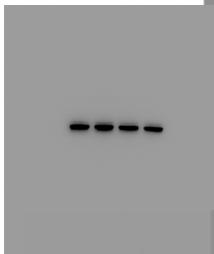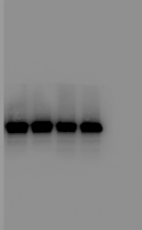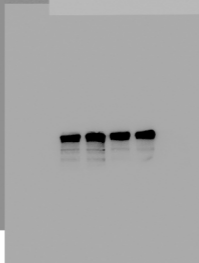

Supplement: Supplementary file 2 — supplementary file WB [file 41420_2025_2384_MOESM2_ESM.pdf]
